# Longitudinal THRIVE study protocol: social and neurobiological mechanisms of risk and resilience in young people with a history of childhood adversity

**DOI:** 10.1136/bmjopen-2026-123790

**Published:** 2026-10-29

**Authors:** Elizabeth E L Buimer, Maximilian König, Pauline Wessels, Hannah Dorsman, Antoinette Haverhals, THRIVE consortium, Augeo Jongerentaskforce, Peter A Bos, Geert-Jan Will, Anne-Laura van Harmelen

**Affiliations:** 1 Leiden University Institute of Education and Child Studies, Leiden, The Netherlands; 2 Leiden Institute of Brain and Cognition, Leiden University Medical Center, Leiden, The Netherlands; 3 Leiden Institute for Brain and Cognition, Leiden University Medical Center, Leiden, The Netherlands; 4 Department of Clinical Psychology, Utrecht University, Utrecht, The Netherlands

**Keywords:** MENTAL HEALTH, Adolescent, Brain, Stress, Psychological, Social Support

## Abstract

**Abstract:**

**Introduction:**

Childhood adversity (CA) is among the strongest predictors of mental health problems. CA refers to experiences that are likely to require significant adaptation by an average child and that represent a deviation from the expectable environment. CA-related alterations in social and cognitive functioning may result in the thinning of social networks and increases in stressful peer interactions which subsequently lead to mental health problems. The Towards Health and Resilience in Volatile Environments (THRIVE) study takes a longitudinal approach to investigate these proposed prospective relationships by examining whether and how social, cognitive and neurobiological mechanisms interact to shape friendship support, stressful peer interactions and mental health functioning over time.

**Methods and analysis:**

The ongoing THRIVE study includes 283 participants aged 18–24 years who retrospectively self-report CA. This longitudinal study comprises six sessions in total. Participants complete an online baseline assessment of mental health, social support, stress experiences and early life experiences, followed by an in-unit deep-phenotyping session. Assessments include self-report measures, cortisol measures, computerised cognitive tasks and functional neuroimaging paradigms assessing stress and social evaluation. Participants subsequently complete four online follow-up assessments at 3-month intervals. Using data collected at the in-unit session, we aim to test whether CA is associated with biased emotion processing, blunted feedback learning and enhanced stress responses, and whether social support from peers may moderate these effects. The longitudinal design further enables us to test whether CA contributes to mental health problems through its impact on cognitive functioning, which over time leads to reduced social support and increased social stress. By elucidating how cognitive and social functioning contribute to mental health outcomes following CA, the THRIVE study aims to inform the development of interventions that foster resilience in young people.

**Ethics and dissemination:**

The THRIVE study was approved by the Medical Ethics Committee Leiden-The Hague-Delft (NL80017.058.21). The study was co-created with the Augeo Youth Task Force, a panel of young adults with lived experience of CA, who contribute throughout the research process. Findings will be disseminated through scientific publications, conference presentations, preprints and public engagement activities.

**Strengths and limitations of this study**

- A large population of 283 young adults with self-reported childhood adversity is included.
- The study includes endocrine, cognitive, neuroimaging and self-report assessments across six consecutive assessment waves.
- The study is co-created with a panel of young people with a history of childhood adversity.
- Childhood adversity is assessed using retrospective self-report, which may induce recall bias.
- Young people with severe depressive or suicidal symptomatology were excluded, potentially limiting representativeness.

## Introduction

 One out of two children and adolescents growing up worldwide are exposed to at least one adverse childhood experience, such as abuse, witnessing domestic violence or substance abuse in the household.^1^ Prevalence estimates of childhood adversity (CA) vary substantially across studies, depending on how adversity is conceptualised and measured, including the types of experiences assessed and the assessment methods used.^2^ CA can be defined as ‘experiences that are likely to require significant adaptation by an average child and that represent a deviation from the expectable environment’ (McLaughlin, p363).^3^ As such, CA refers to a wide range of adverse childhood experiences including child maltreatment (emotional, sexual and physical abuse and emotional and physical neglect) as well as family adversity (eg, marital distress/conflict, parental alcohol dependence, aggressive parenting behaviour, parental violence, parental mental health problems or stressful family-level life events).^4^ The present study operationalises CA as a broad construct encompassing abuse and neglect as well as other adverse experiences, such as parental separation and the loss of a loved one.

CA is a strong transdiagnostic predictor of mental health problems in adolescence, such as depression, anxiety and externalising problems.^5–8^ Furthermore, CA is associated with an unfavourable course of mental health problems, including earlier onset of psychopathology and more severe, persistent symptomatology.^9 10^ Different forms of CA frequently co-occur, creating cumulative risk for the development of mental health problems.^11^ Dose–response relationships between CA and mental health have been widely reported,^7 12 13^ with experiences related to maladaptive family functioning posing the strongest risk.^6 7 14^

When studying associations between CA and (mental) health outcomes, CA can be operationalised either as the number of distinct forms experienced (cumulative model^11 15^) or as separate dimensions with dimension-specific effects (dimensional model^16^). Dimensional models of adversity suggest that abuse and neglect capture differential experiences with distinct impacts on cognition and the brain. Experiences of abuse comprise (threat of) harm to a child—whereas neglect involves deprivation leading to the absence of developmental stage salient expected inputs from the environment needed for normative development. Preliminary support for dimensional models of CA suggests that experiences of threat may lead to higher sensitivity to threat signals and more rapid identification of emotional stimuli, whereas experiences of deprivation may impact higher-order cognitive functioning, such as our ability to learn from positive and negative feedback.^17 18^ Recently, unpredictability was conceptualised as a potential third dimension of adversity.^19–22^ Cumulative risk models suggest that discrete forms of adversity do not have distinct but additive effects on cognitive functioning, with more adversity being associated with stronger but not necessarily specific effects. According to this model, investigating the number rather than the nature of CA experiences may thus have greater translational value and clinical relevance.^23^

Studies using dimensional or cumulative models of CA provide important evidence that CA affects cognitive functioning across multiple domains, with the strongest support for heightened threat processing, reduced reward processing and altered emotion regulation.^17 24^ First, individuals with a history of CA are more sensitive to threat, evidenced by faster recognition of angry and fearful faces, while happy faces prove more difficult to recognise.^25 26^ These social information processing biases are linked to externalising problems^27 28^ and internalising problems in maltreated children,^29 30^ although no effect on internalising symptomatology was found in one study.^28^ Second, CA is consistently associated with impaired reward learning and, to a lesser extent, reward valuation,^31^ which may represent a pathway to later depressive symptoms.^32^ Childhood experiences of deprivation may be particularly linked to blunted reward learning, as reduced exposure to responsive caregiving limits early learning based on the rewarding value of social feedback,^16^ potentially impairing future social feedback learning. Finally, CA is associated with emotion-regulation difficulties, including a reduced ability to modulate emotional responses and greater reliance on strategies linked to poorer mental health.^17 33 34^ Additionally, there is growing evidence for changes in autobiographical memory.^35–38^ Young people with CA may have more difficulties recalling specific details of autobiographical memories and negative memories can be more prominent than positive memories and these changes in autobiographical memory have been associated with depression.^35 37 39^ Together, these converging alterations in threat processing, reward processing, emotion regulation and autobiographical memory may represent interrelated pathways through which CA can influence later mental health.

Integrating empirical findings, contemporary developmental frameworks outline mechanisms through which adversity-related neural and behavioural alterations may shape mental health trajectories, with effects moderated by individual differences and protective factors. According to the ‘theory of latent vulnerability’,^24^ cognitive alterations related to childhood adversity may be adaptive in the short-term, while reducing mental health in later life or in contexts with less adversity.^24^ The ‘neurocognitive social transactional model of psychiatric vulnerability’^40^ builds on the theory of latent vulnerability. This framework suggests that individuals with CA may carry latent vulnerabilities that can generate social stress (ie, ‘stress generation’) and reduce social support over time (ie, ‘social thinning’). Stress generation and social thinning in turn are thought to increase risk for developing mental problems over time. According to this model, it may be adaptive in a stressful home environment to rapidly detect angry facial expressions, to expect negative feedback and to adapt behaviour and emotions accordingly. Over time, or in different (less stressful) social environments, expecting negative feedback and biased emotion processing may, however, inadvertently and unintentionally increase the likelihood of misunderstandings and problems with peers (eg, a smile may be misattributed as being laughed at, a friendly gesture may be missed or ignored). Indeed, there is strong empirical support that CA is associated with biased emotion processing and blunted feedback learning^3 18 41 42^ and difficulties in building or maintaining social relationships.^43–45^ Indeed, adolescents with CA are more likely to experience low friendship support^46 47^ and are more sensitive to psychosocial stress from peers.^48^ However, most of the existing empirical support for the social transactional model is cross-sectional, limiting inferences about temporal ordering and directionality. Still, two recent studies, both conducted during the COVID-19 pandemic, showed an association between CA and social thinning across multiple timepoints.^49 50^

Fortunately, not all individuals with CA develop mental health problems. A substantial proportion of individuals show resilience at one or more stages of their lives. Resilience refers to the capacity to maintain or regain mental health despite exposure to adversity.^51 52^ In the context of CA, such resilience can be inferred from psychosocial functioning at any given point in time, that is better than expected or better than that of others with similar CA experiences.^53 54^ Social support plays an important role in aiding such resilient mental health functioning after CA.^53 55 56^ Friendships, defined as voluntary, reciprocal and nurturing relationships, may be a particularly important source of social support for young people.^57^ We have shown that friendship support improves resilient functioning after CA, especially in adolescents and young adults.^46 49 53 55 58^ While the exact mechanisms through which social support protects against mental health problems are currently unknown,^47^ social stress buffering models suggest that the presence and availability of a social partner reduce the reactivity to threat in biological stress systems.^59^

To aid intervention and prevention studies, the longitudinal Towards Health and Resilience in Volatile Environments (THRIVE) study will prospectively assess mental health, cognitive functioning, endocrine responses, brain function and social experiences in 288 young adults aged 18–24 years. In doing so, the THRIVE study will test all components of the neurocognitive social transactional model of psychiatric vulnerability^40^ in one cohort over time. As such, THRIVE will elucidate whether and how changes in cognitive functioning related to CA may contribute to reduced friendship support and heightened social stress over time. Furthermore, using a well-validated stress-induction, the THRIVE study will examine social stress buffering models^59^ to examine whether friendship support reduces immediate stress perceptions and neurobiological stress responses. Finally, we expect to provide new insights on the dynamics of resilience after CA. Specifically, given the longitudinal assessments of stress, we can investigate predictors and mechanisms of resilience to CA as evoked by new stressors in daily life.

The THRIVE study has four objectives (figure 1). As a first objective, we will compare two competing hypotheses regarding the effects of CA on emotion processing and social feedback learning. Specifically, we will investigate whether associations with these outcomes are best explained by distinct effects of threat and deprivation experiences (dimensional approach) or by equivalent, non-specific effects across adversity dimensions (cumulative risk approach). Based on the dimensional model,^16^ we expect that threat experiences predict biased information processing towards threat cues in an emotion processing task but not blunted social feedback learning. In contrast, deprivation experiences would result in blunted social feedback learning but not biased information processing. Support for the competing cumulative risk account would be found when threat *and* deprivation experiences show similar effects on both emotion processing and feedback learning outcomes, suggesting that the overall burden of adversity is more important than the specific nature of adversity exposure. As a second objective, we will test the social stress buffering hypothesis that social support moderates the effect of CA on neural stress reactivity using a cross-sectional design.^47 58^ In the third objective, we will use the longitudinal assessments of mental health, social stress and social support to empirically test whether CA-related cognitive alterations lead to stress generation and social thinning, as described in the social transactional model.^40^ To do so, we will investigate to what extent emotion processing and feedback learning in an experimental setting predict stress generation and social thinning outside the controlled lab environment. We hypothesise that more pronounced emotion processing biases (towards threat) will predict more stressful life experiences and lower friendship quality at baseline as well as a more rapid worsening of both over time. Furthermore, we hypothesise that blunted social feedback learning will predict more social stress and lower social support as well as a steeper worsening of both over time. In the fourth objective, we will model the dynamics of friendship support, stress experiences and mental health at the intra-individual level to determine whether within-person decreases in friendship quality or increases in social stress impact on later mental health. We hypothesise that a within-person decrease in friendship quality will be related to more stressful events and reductions in mental health.

**Figure 1:**
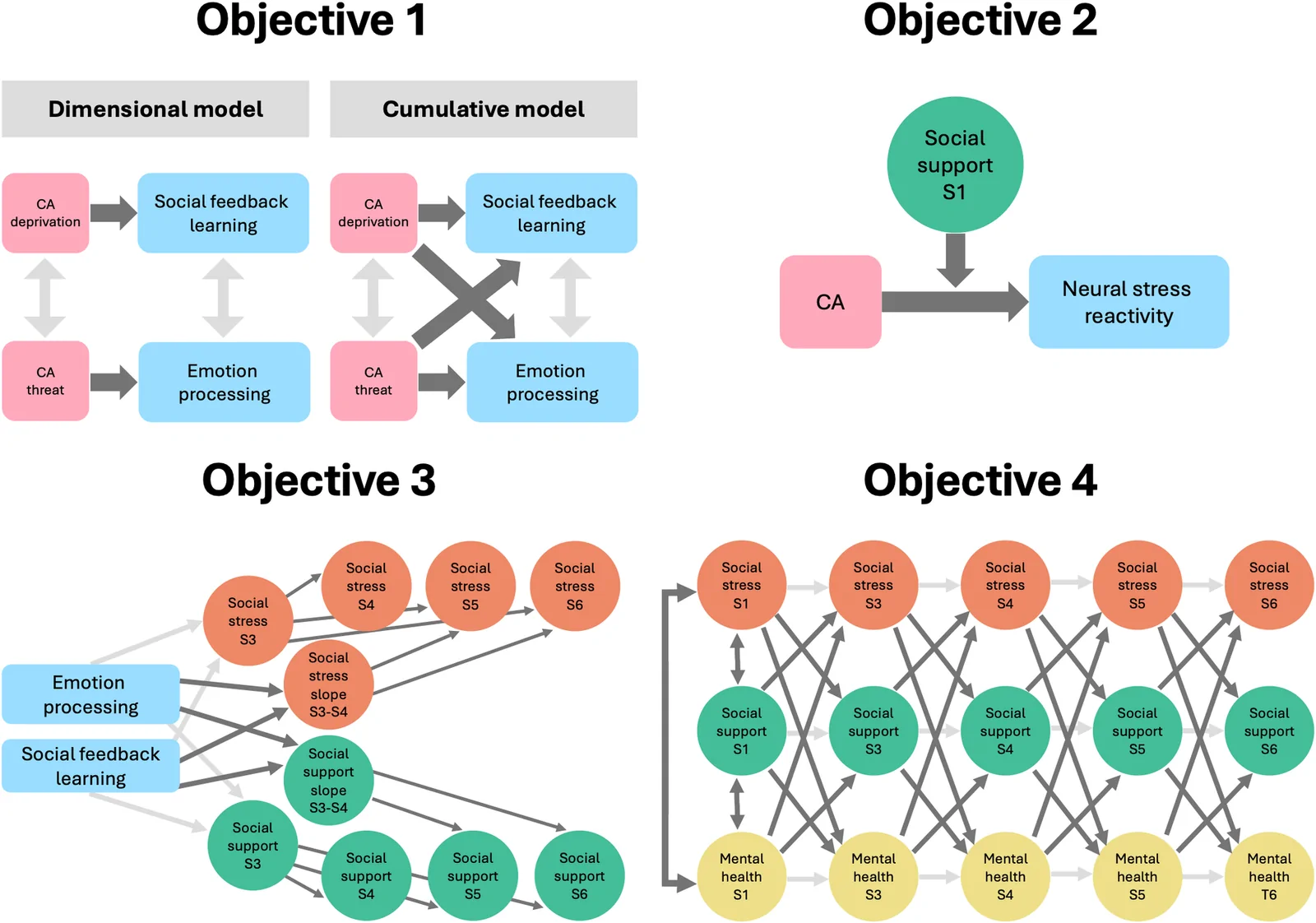
The main objectives of the Towards Health and Resilience in Volatile Environments (THRIVE) study. The THRIVE study tests four objectives: (1) using path modelling and model comparisons to evaluate evidence for dimensional and cumulative models of childhood adversity (CA). Model comparisons will be used to examine whether threat and deprivation have distinct effects on emotion processing (threat bias) and social feedback learning (blunting). Distinct effects would support dimensional models of CA, whereas equivalent effects would support cumulative risk models. (2) Social stress buffering hypothesis: testing whether social support moderates the association between CA and neural stress reactivity. (3) Social transactional model: using longitudinal assessments (S1 to S6) to evaluate whether CA-related alterations in emotion processing and feedback learning predict stress generation and social thinning (more stressful experiences, lower social support and steeper worsening over time). (4) Within-person dynamics: modelling intra-individual coupling between friendship quality, social stress and mental health, hypothesising that within-person reductions in friendship quality and increases in social stress precede declines in mental health. S1 to S6 refer to the different sessions at which the repeated measures are collected.

## Methods and analysis

### Sample recruitment and eligibility

We aim to recruit N=288 young adults. Participants are being recruited through flyers and posters at schools, general practitioner practices, libraries, hospitals, sport centres, outpatient care facilities, social media, websites, university recruitment sites and more. To be eligible for the THRIVE study, participants need to be between 18 and 24 years during the telephone screening. During that screening, participants should self-report at least one adverse experience before the age of 18, assessed using the Youth and Childhood Adversity Scale.^60^ Furthermore, participants must pass the MRI-safety screening and be able to understand, write and speak Dutch as the questionnaires and instructions are in Dutch. Given that the study protocol can be perceived as stressful, particularly for adolescents experiencing elevated levels of emotional distress, we used the 9-item version of the Patient Health Questionnaire^61^ to assess severe depressive symptoms or suicidal ideation over the past 2 weeks, rated on a Likert scale from 0 (not at all) to 3 (nearly every day). Young people were excluded from participation if their total score was higher than 14 or when they scored higher than 0 on the suicidal ideation item (‘Thoughts that you would be better off dead or of hurting yourself in some way’). Other exclusion criteria were factors that may confound the interpretation of the neuroimaging data, such as a history of significant head trauma, premature birth, impaired vision or colour perception and daily use of medication to treat symptomatology related to neurodevelopmental diagnoses.

The first participant was enrolled on 4 October 2022 and the last participant was enrolled on 15 April 2026, resulting in a final sample of 283 participants. Data collection is ongoing, with the final follow-up assessments scheduled for completion in May 2027.

### Procedure

This study consists of a telephone screening, one online assessment (session 1), one in-unit assessment day at the Leiden Institute of Brain and Cognition at the Leiden University Medical Center (session 2) and four online follow-up assessments (sessions 3, 4, 5 and 6) at 3-month intervals. Figure 2 provides an overview of the study procedure and the constructs assessed at each session. The order of the task and questionnaires is the same for all participants.

**Figure 2:**
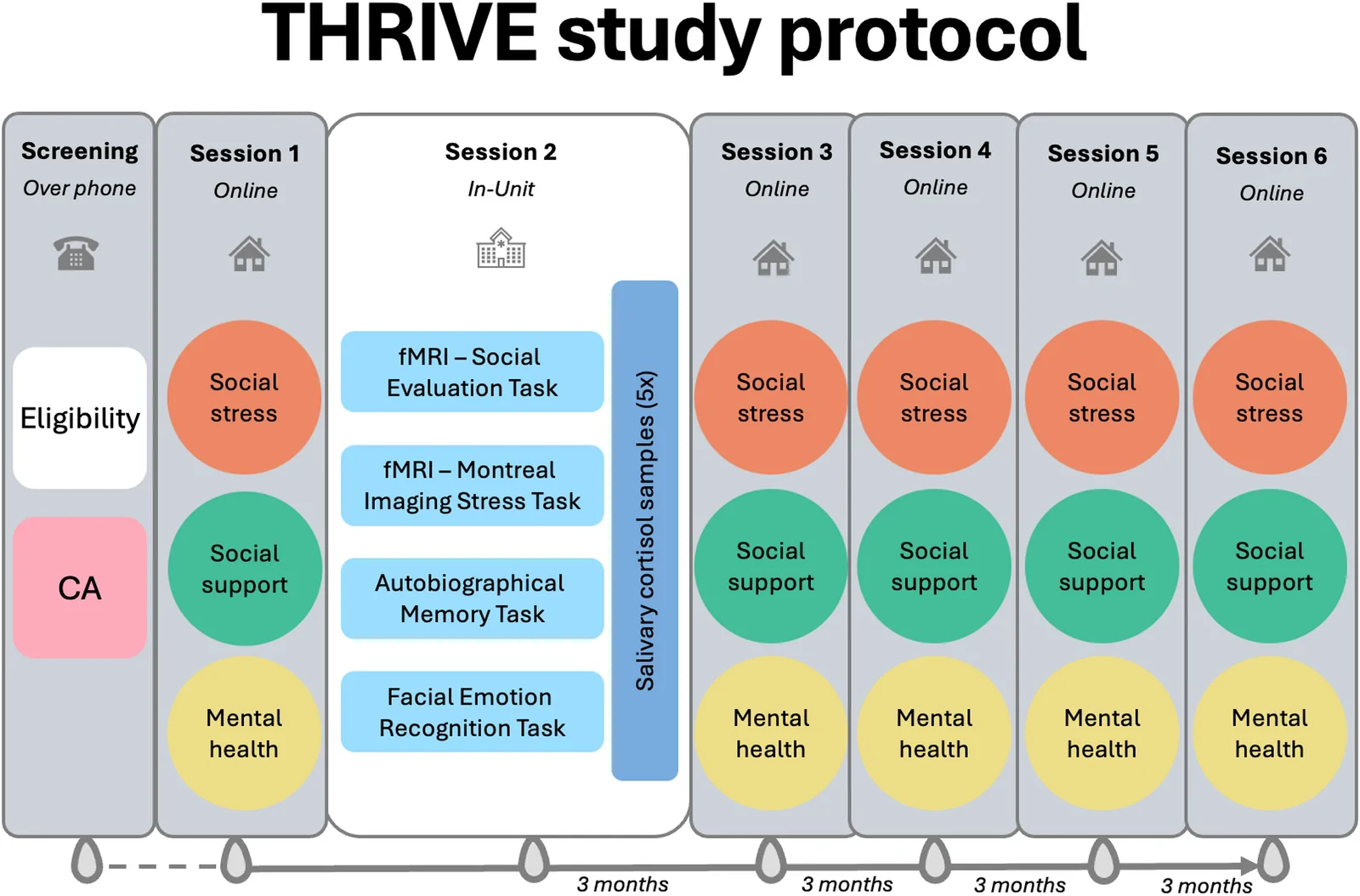
The design of the Towards Health and Resilience in Volatile Environments (THRIVE) study. In the THRIVE study, we aim to recruit 288 young adults with self-reported childhood adversity (CA). After prospective participants sign up, a member of the research team calls them to screen for eligibility, including the presence of at least one adverse childhood experience. Eligible participants first complete an online questionnaire battery at home (session 1) assessing social stress, mental health and social support. Next, they attend a 4-hour in-unit visit (session 2), during which neuroimaging and cognitive task data are collected alongside additional questionnaires. The MRI protocol begins with the Social Evaluation Task, followed by an anatomical scan and the Montreal Imaging Stress Task. Across the testing day, five salivary cortisol samples are collected: three before and two after the Montreal Imaging Stress Task. The Autobiographical Memory Task and the Facial Emotion Recognition Task are administered outside the MRI scanner. The in-unit visit concludes with a debriefing. After session 2, participants are invited to complete follow-up questionnaires (sessions 3–6) repeating the measures from session 1 every 3 months for 1 year. fMRI, functional MRI.

### Data collection

#### Questionnaire data

Table 1 lists the questionnaires used in the THRIVE study, the time points at which they are administered, and the constructs they assess. Further details on the questionnaires are provided in online supplemental file. In addition to the questionnaires shown in table 1, demographic information is collected, including date of birth, sex assigned at birth, gender identity, ethnic identity, sexual orientation, highest level of education, occupation, parental household income and parental education level. The selection of these variables was informed by previous work.^62^

**Table 1:** Questionnaires in the Towards Health and Resilience in Volatile Environments (THRIVE) study including description, reference and measurement wave

Questionnaires	Session
Childhood adversity	
Youth and Childhood Adversity Scale (YCAS)—severity of different types of adverse experiences^60^	0
Childhood Trauma Questionnaire (CTQ)—severity of different types of childhood trauma, producing five clinical subscales: emotional abuse, physical abuse, sexual abuse, emotional neglect and physical neglect^90 91^	1
Mental health	
Patient Health Questionnaire (PHQ)—level of depressive symptoms^61^	0, 1, 2, 3, 4, 5, 6
State-Trait Anxiety Inventory (STAI)—level of trait and state anxiety^92^	1, 2, 3, 4, 5, 6
Mood and Feelings Questionnaire (MFQ)—level of depressive symptoms^93^	1, 2, 3, 4, 5, 6
Drugs, Alcohol and Self-Injury Inventory (DASI)—cigarette, alcohol, drug use and non-suicidal self-injury^94^	2
Psychiatric History Self-Report Questionnaire (PHSQ)—history of current and lifetime psychiatric diagnoses and current treatment	2*
Social support	
McGill Friendship Questionnaire–Friendship Functions (MFQ-FF)—perceived friendship support across six subscales: stimulating companionship, help, intimacy, reliable alliance, self-validation and emotional security^95^	1, 3, 4, 5, 6
Multidimensional Scale of Perceived Social Support (MSPSS)—social support across three domains: friends, family and significant others^96^	1, 3, 4, 5, 6
Cambridge Friendship Questionnaire (CFQ)—number, availability and quality of friendships^46 53^	1, 3, 4, 5, 6
Strengths and Difficulties Questionnaire (SDQ)—subscale prosocial behaviour^97 98^	1, 3, 4, 5, 6
Social stress	
Multidimensional Peer Victimisation Scale (MPVS)—peer victimisation with six subscales: physical victimisation, verbal victimisation, social manipulation, attacks on property, electronic victimisation, social rebuff^99^	1, 3, 4, 5, 6
Life Events Questionnaire (LEQ)—recent life events that occurred in the past 3 months and perceived impact of each event^100–105^	1, 3, 4, 5, 6
Strengths and Difficulties Questionnaire (SDQ)—subscale peer problems^97 98^	1, 3, 4, 5, 6
Perceived Stress Scale (PSS)—level of perceived stress^106^	1, 2, 3, 4, 5, 6
Personal traits and self-esteem	
The Big Five Inventory-2 (BFI-2)—extraversion and negative emotionality included^69^	1
Rosenberg Self-Esteem Scale (RSES)—positive and negative feelings of self-worth^107^	1
Hypomanic Personality Scale (HPS)—traits of an overactive, gregarious personality style that has been shown to correlate with emotional instability^108^	1
Other	
Social Media Use Questionnaire (SMUQ)—time spent and views on social media^109^	1
Coronavirus Impact Scale (CIS)—impact of the pandemic on personal life^110^	2
Edinburgh Handedness Inventory–Short Form (EHI-S)—dominant hand for various activities^111^	0

* The Psychiatric History Self-Report Questionnaire was introduced later in the study. Participants who had already completed session 2 received the questionnaire retrospectively.

#### Behavioural data

##### Facial Emotion Recognition Task

The Facial Emotion Recognition Task was developed for this study to measure accuracy and speed of emotion labelling. We are currently collecting construct validity evidence by examining correlations between performance on our task and an established emotion recognition task. Our task begins with a calibration phase to test participants’ reaction time independent of emotion processing. In this phase, participants press the spacebar when they see an icon of a cat. In the main task, participants view short movie clips in which a facial expression either remains neutral or changes from neutral to sad, happy, angry or fearful over approximately 6 s (figure 3). The participant is instructed to press the spacebar to stop the clip as soon as they recognise the expressed emotion. Pressing the spacebar is used to compute emotion labelling speed, and the subsequent answer to a forced-choice labelling question was used to compute emotion labelling accuracy.

**Figure 3:**
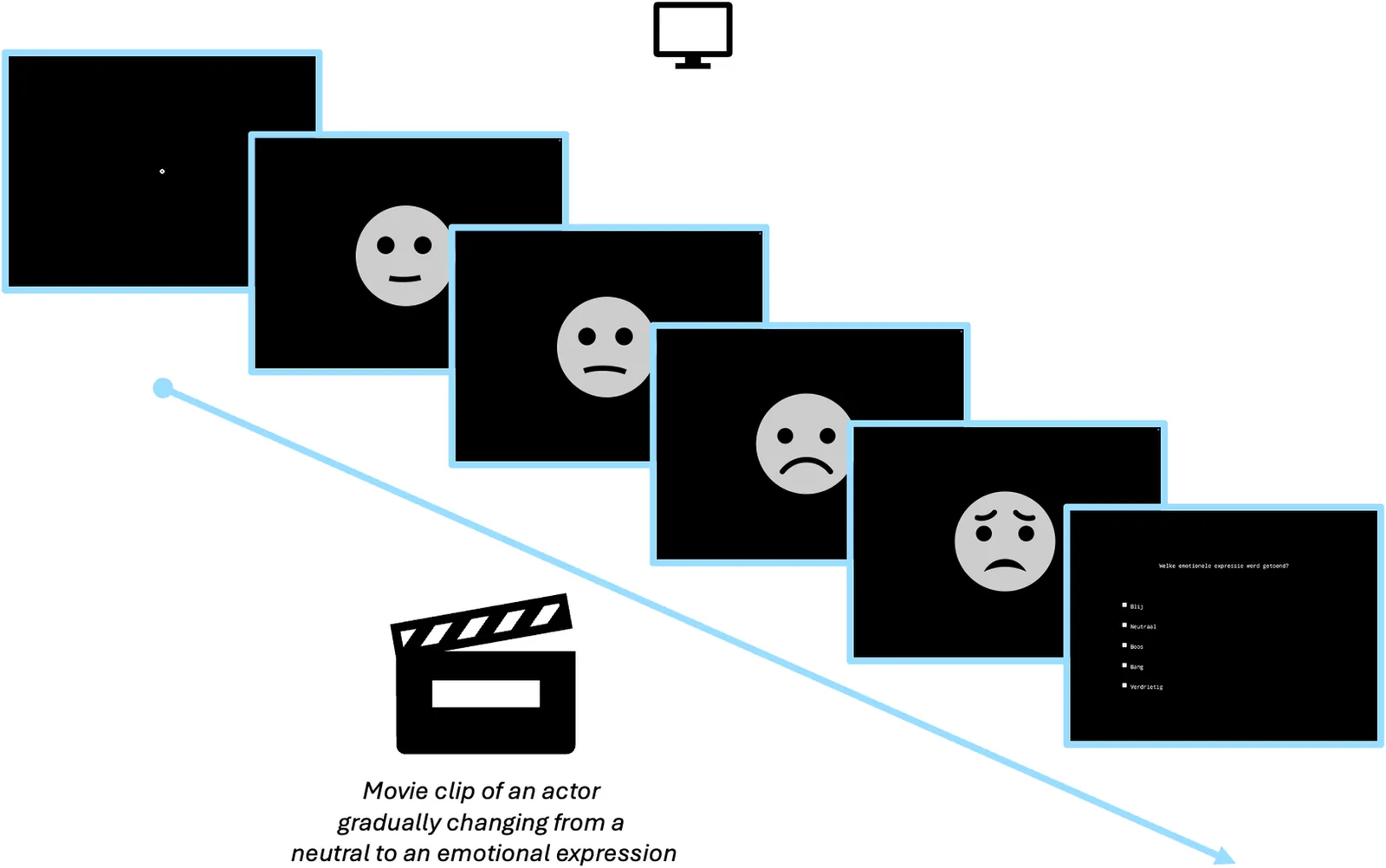
Design of the Facial Emotion Recognition Task. During the Facial Emotion Recognition Task, participants view short movie clips of an actor whose facial expression either remains neutral or gradually changes from neutral to sad, happy, angry or fearful over approximately 6 s. As soon as the participant recognises the emotion, they stop the clip by pressing the spacebar (reaction time) and answer a forced-choice labelling question (accuracy). A selection of movie clips from the Amsterdam Dynamic Facial Expression Set (ADFES) was used.^63^ For privacy and in accordance with BMJ image and anonymisation requirements, the original facial stimuli are represented by schematic emoticons in this figure. Examples of the original ADFES stimuli can be found in^63^ and on the ADFES website. MIST, Montreal Imaging Stress Task.

Stimuli are drawn from the Amsterdam Dynamic Facial Expression Set.^63^ The experimental set consists of 20 faces (10 female, 10 male) from different ethnic backgrounds. Before the experimental trials, participants complete 10 practice trials using stimuli from two male actors not included in the main set. Five types of facial expressions are used: neutral, sad, happy, angry and fearful. Stimuli are presented against a black background in a random order. Each trial begins with a white circle on a black background presented for 500 ms. The experiment consists of 100 randomised experimental trials, preceded by 10 practice trials. The task was programmed in OpenSesame^64^ and is displayed on a 19-inch monitor with a 60 Hz refresh rate and 1024×768 resolution.

##### Autobiographical Memory Task

Autobiographical memory specificity is assessed using an adapted written, computerised version of the Autobiographical Memory Task^65^ focused on friendship memories. At the in-unit session, participants are presented with four positive and four negative Dutch cue words in the following fixed order: *gelukkig* (happy), *boos* (angry), *leuk* (nice), *jaloers* (jealous), *grappig* (funny), *gekwetst* (hurt), *gezellig* (cosy), *eenzaam* (lonely). For each cue, participants are given 2 min to describe a friendship memory that the word reminded them of, with the instruction to recall different, real memories that could be either recent or from years ago. Minimal instructions are given to avoid biasing specificity and allow for maximal inter-individual differences, a procedure shown to increase measuring sensitivity in non-clinical samples.^66^

### Neuroimaging data

#### Acquisition

Structural and functional MRI scans are acquired on a Philips Achieva dStream 3T (Philips Medical Systems, Best, The Netherlands), using a 32-channel head-coil. Whole-brain, T2*-weighted echo planar images (EPIs) are acquired during the Social Evaluation Task (SET) and subsequently during the Montreal Imaging Stress Task (MIST). The EPIs are acquired using the following parameters: repetition time (TR) =2200 ms; echo time (TE) =30 ms; flip angle=80°; 2.75 mm×2.75 mm in-plane resolution; 2.75 mm slice thickness; 40 slices per volume; SENSE factor 2.5 (anterior–posterior). The SET consists of two blocks of maximum 370 dynamic scans each. The MIST consists of three blocks of maximum 215 dynamic scans each. The number of dynamic scans will differ between individuals for both tasks depending on the response time of the individual. Two times during the MRI protocol, that is, after the SET and after the MIST, a B0-map is acquired (TR=200 ms; TE=shortest default; flip angle=30°; voxel size=2.75 mm×2.75 mm×2.75 mm). The B0-map will be used to correct for distortions due to B0 inhomogeneities when pre-processing the EPIs. For anatomical reference a structural T1-weighted 3D gradient echo scan is acquired with the following parameters: TR=7.9 ms; TE=3.5 ms; flip angle=8°; voxel size=1.1 mm×1.1 mm×1.1 mm. Tasks are displayed on a 32-inch monitor (CRS BOLDscreen32).

#### Functional MRI: SET

Social feedback learning will be measured with a modified version of a previously validated SET.^67 68^ Participants receive computer-generated approval (‘likes’) or disapproval (‘dislikes’) from ostensibly real peers in response to an online personal profile the participants created before the testing day (figure 4). Each peer is represented by a name and a coloured icon associated with a specific probability of giving positive feedback (87%, 67%, 33%, 13%). On each trial, participants can predict whether a peer liked or disliked them before receiving their like or dislike. After every second to third trial, participants rate their current self-esteem on a visual analogue scale (‘How good do you feel about yourself right now?’).

**Figure 4:**
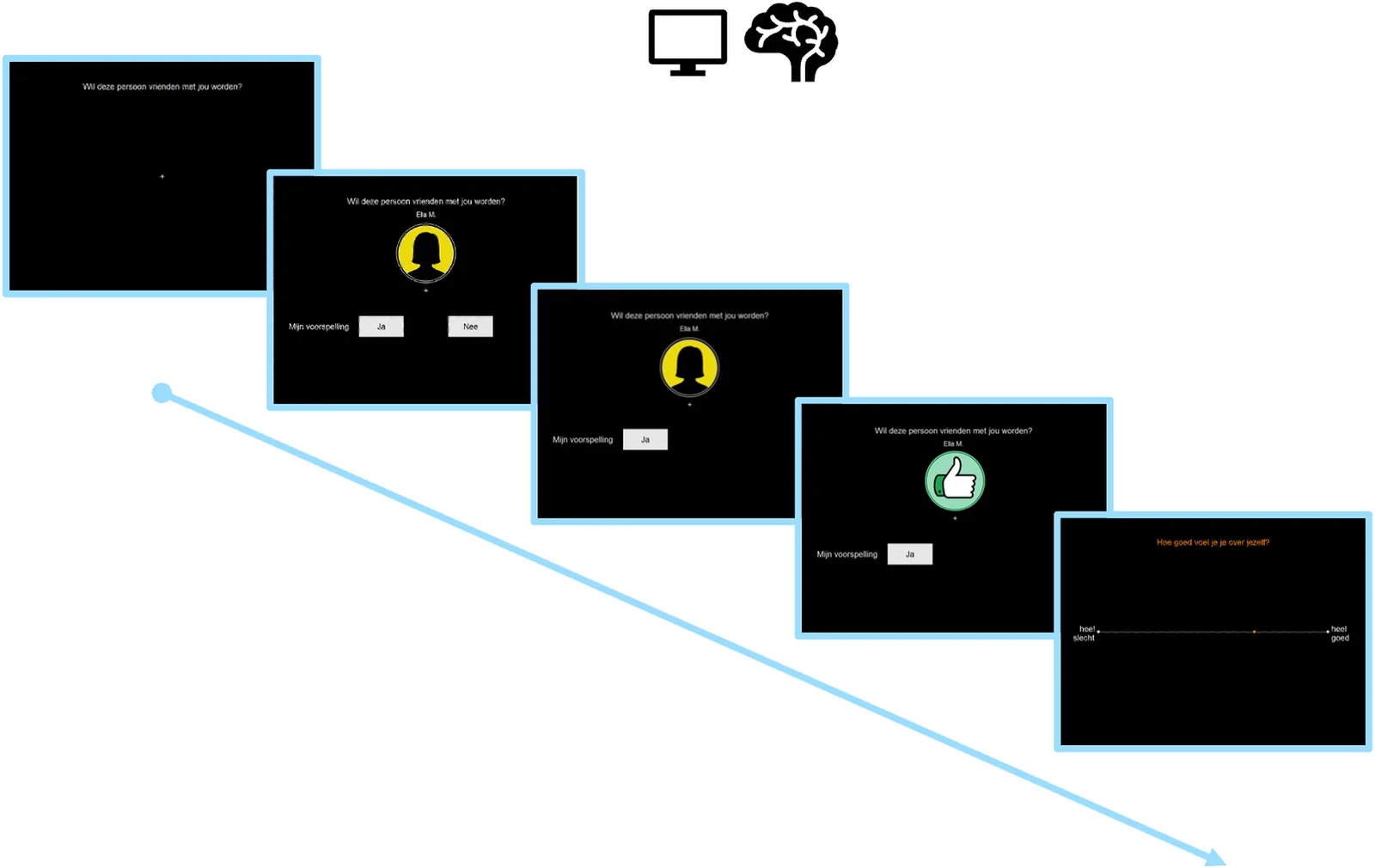
Design of the Social Evaluation Task. The Social Evaluation Task^67 68^ is administered in the MRI scanner while collecting functional neuroimaging data. Participants predict whether a rater would want to befriend them based on a personal profile they created earlier. The background colour of each rater is related to the probability of receiving a like or dislike. After each trial, participants receive a like or dislike that could either match or mismatch their prediction. By learning from the colour cue participants could improve their prediction accuracy over time. After every 2–3 trials participants indicate their level of self-esteem on a visual analogue scale.

The participants first practice the task outside the MR scanner. After practicing, the experimental task within the MR scanner consists of two runs. Between runs a self-esteem manipulation is administered to be able to investigate the effect of self-esteem on social feedback learning and related neural activity. Participants are randomly assigned (1:1) to a positive (‘boost’) or negative (‘diminish’) condition. Between the two runs, participants first receive veridical feedback about their level of extraversion based on their answers to the Big Five Inventory-2.^69^ Subsequently, depending on the condition they are in, they receive a message meant to boost or to diminish their self-esteem (based on the future alone and future belonging conditions in earlier work^70–73^). In the boost condition, participants are told that based on their personality they would have rewarding relationships throughout life and in the diminish condition they are told that they based on their personality they would likely end up alone. This life-alone paradigm is a commonly used method to manipulate self-esteem with a meta-analysis showing a moderate effect size.^74^ After the self-esteem manipulation between the two runs is completed, the second run introduces new peer groups with different colour-coded icons to reset learning and prevent carryover effects. After this run, participants select their preferred rater. Next, they receive a positive message ostensibly from that rater, referencing their self-created profile and expressing they would want to befriend them based on the personal details in the profile.

This design allows us to examine neurocomputational mechanisms supporting social learning and self-esteem, both at baseline and in an experimentally induced high or low self-esteem state. To ensure no participant finishes the task with reduced self-esteem, those in the negative condition receive five additional ‘likes’ at the end of the second block, which will not be analysed. At the end of the testing day, participants are fully debriefed, explaining that all feedback was computer-generated and not based on actual peer evaluations and that the prediction of future social relationships was randomly assigned and not based on their answers to self-report questionnaires.

#### Functional MRI: MIST

Neural stress responses will be examined using the MIST^75^, a validated functional MRI paradigm in which participants are asked to solve arithmetic problems that are too difficult under time pressure.^76^ The protocol includes a practice session outside the MR scanner to familiarise with the task, a practice session in the scanner and an experimental session in the scanner. The task is administered on the Inquisit Millisecond platform.

During the practice session in the scanner, participants’ average response times for arithmetic problems at varying difficulty levels are recorded to set individualised time limits for the experimental blocks. During the experimental block, the time limit is set 10% below the participant’s average response time to induce a high failure rate (figure 5). The programme dynamically adjusts the time limit based on performance. The average accuracy is set to a fictional 90%. Between runs, participants receive verbal feedback from a study team member to further induce stress, for example, ‘You are not answering enough of these correctly. Please try as hard as you can’. Previous studies show that this combination of different stressors (time pressure, performance pressure, social comparison, verbal feedback) reliably induces stress.^77 78^

**Figure 5:**
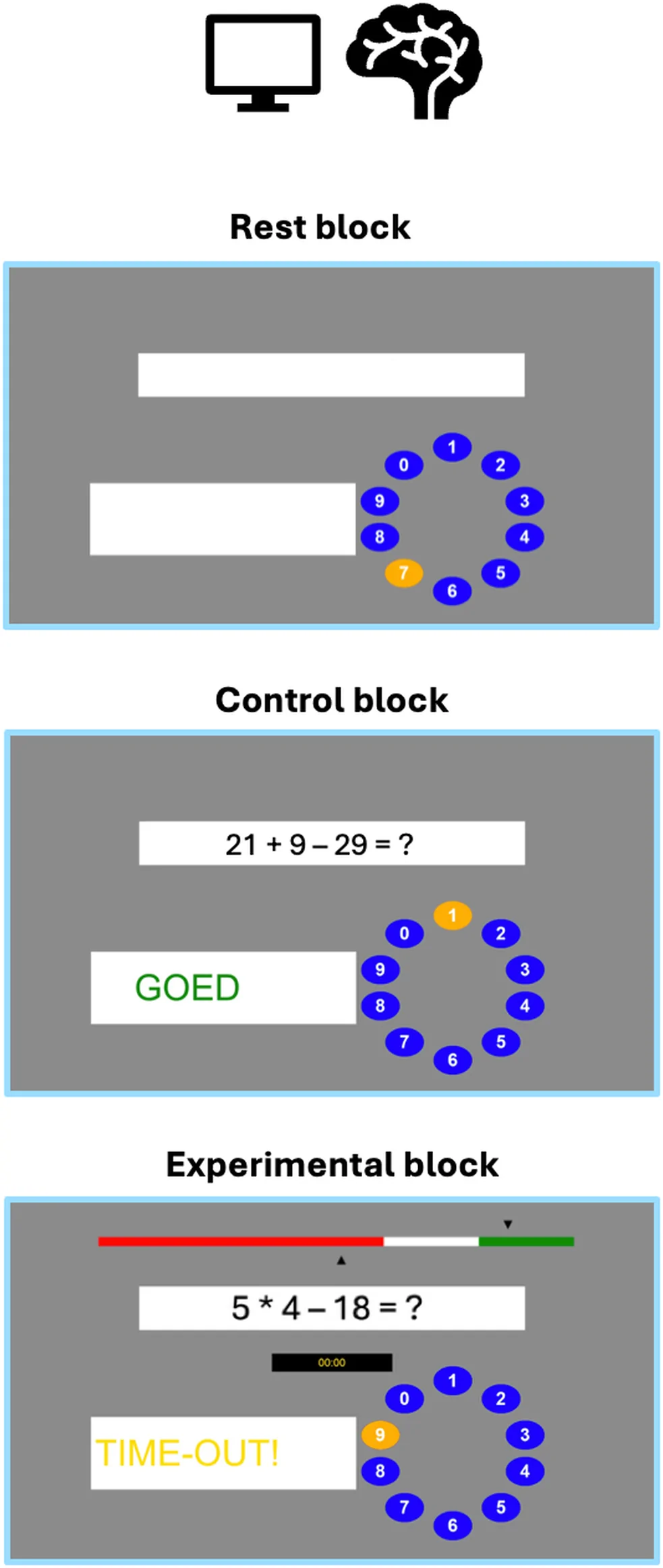
Design of the Montreal Imaging Stress Task. The Montreal Imaging Stress Task^75^ is administered in the MRI scanner while collecting functional neuroimaging data. The task consists of three block types—control, experimental and rest—presented in a semi-random order. During the rest block, participants passively view the screen. In the control block, participants solve arithmetic problems by selecting the correct answer using a response wheel and receive performance feedback. The experimental block is structurally similar but includes a time constraint. In addition, participants are shown a comparison between their own average performance and a very high (fictitious) group-level accuracy.

The task comprises three runs, each including a control, experimental, and rest block presented in semi-randomised sequences. In the control block, task difficulty and frequency match the experimental block, but there is no time restriction, and average performance is not displayed; feedback is limited to ‘correct’ or ‘incorrect’. During the rest block (~2 min), the interface remains on screen, but no arithmetic problems are presented.

Self-reported mood (visual analogue scale) and salivary cortisol are measured before and after the MIST to verify that the task induces a subjective and hormonal stress response. To be able to control for individual differences in nonverbal reasoning, we administer the Matrix Reasoning subtask of the Wechsler Adult Intelligence Scale IV-NL post-scanning.^79^ Overall, our design ensures precise assessment of neural, behavioural and hormonal responses to psychosocial stress while controlling for individual differences in arithmetic ability and task performance. At the end of the testing day, participants are fully debriefed in person to explain every task manipulation used to induce stress.

### Cortisol measures

Salivary cortisol concentration will be collected with Salivette collection devices three times before and two times after the MIST. Up until the last cortisol assessment, participants are asked to not consume any food or drinks. After the last sample is collected, participants are offered snacks and drinks. Saliva sampling commences approximately 20 min after participants arrive on the in-unit testing day (ie, ~130 min prior to the MIST). A second sample is collected 30 min later. Subsequent samples are taken immediately before the MIST and 5 and 40 min after the MIST. Saliva samples will be stored in freezers and shipped in batches to Dresden, Germany, where free cortisol levels will be measured using a chemiluminescence immunoassay at the Kirschbaum Lab.

### Data analysis

#### Data reduction methods to estimate main constructs

To estimate our main constructs (ie, CA, social support, social stress and mental health), we will use principal component analyses (PCAs) as a data reduction technique. Separate PCAs will be conducted for each core construct, using item-level data from the questionnaires that assess that specific construct. This approach allows us to derive component scores that summarise patterns of covariation across items measuring the same underlying construct. For CA, the Childhood Trauma Questionnaire was included to provide a detailed assessment of abuse and neglect within the family context, while the Youth and Childhood Adversity Scale was used to capture a broader range of adverse childhood experiences occurring both inside and outside the family context. Items from both questionnaires will be entered into the PCA for CA, thereby accounting for covariance between items across the two measures.

To compare dimensional and cumulative risk accounts (objective 1), we will derive threat and deprivation scores based on theoretically defined adversity categories. Consistent with previous work,^80 81^ deprivation will be indexed by emotional and physical neglect experiences, whereas threat will be indexed by emotional, physical and sexual abuse experiences. These threat and deprivation scores will then be included and using path modelling we will then evaluate their relations with neurocognitive functioning using model comparisons. In the dimensional path-model, threat and deprivation will be allowed to show distinct associations with emotion processing and social feedback learning outcomes. In the cumulative risk path-model, paths from threat and deprivation to the outcomes will be constrained to be equal, reflecting the hypothesis that adversity dimensions exert comparable, non-specific effects. Model fit indices and parsimony criteria will be used to determine which theoretical account better explains the data.

#### Residual approach to quantify resilient functioning

Resilient functioning will be quantified as the degree to which an individual functions better or worse than expected given their self-reported history of CA.^53^ For that, we will use PCAs that reflect current psychosocial functioning across various domains (ie, assessed with the drugs, alcohol and Self-Injury Inventory, Mood and Feelings Questionnaire, State-Trait Anxiety Inventory, Patient Health Questionnaire) as well as a single component that reflects general CA experiences (assessed with the Childhood Trauma Questionnaire and Youth and Childhood Adversity Scale). Individual degree of resilient functioning will then be estimated as the residual variation from the best fitting relationship (comparing linear, quadratic and cubic models) between our component for psychosocial functioning and our component for CA. As such, in our approach resilient functioning reflects the degree to which psychosocial functioning across domains is better (or worse) than expected given an individual’s CA experiences. Using this method, individual residual variation scores will be extracted to indicate individual degree of resilient functioning as compared with the overall sample. An extensive discussion of the benefits and pitfalls of using this approach to quantify resilient functioning after CA can be found in earlier work.^54^

#### Processing of neuroimaging data

Functional MRI data will be pre-processed using widely-used methods such as Statistical Parametric Mapping 12^82^ and Harmonised Analysis of Functional MRI pipeline.^83^ Images will be corrected for movement artefacts, co-registered with the T1-weighted images, normalised to a standard space and spatially smoothed. Quality control will be performed after each preprocessing step. Anatomical MRI will be analysed using FreeSurfer (freesurfer.net^84^). Neuroimaging analyses will be performed using the compute resources from the Academic Leiden Interdisciplinary Cluster Environment provided by Leiden University.

#### Statistical modelling

For objective 1, we will directly compare dimensional and cumulative risk models of adversity using a path modelling framework. Path modelling is a type of structural equation modelling that allows us to simultaneously estimate paths between multiple predictors and outcomes, and to select the best model balancing explanatory power and parsimony. For objective 2, we will use multiple regressions with CA, social support and their interaction as predictors for social feedback learning, autobiographical memory specificity and neural stress responses. For objective 3, we will use growth curve modelling to assess whether increased emotion processing biases and blunted feedback learning are associated with less favourable trajectories of friendship and social stress scores over time. For objective 4, we will use random-intercept cross-lagged panel models across the five repeated measures to determine whether within-person decreases in social support or increases in social stress affect later mental health.

### Patient and public involvement

In this study, we consulted the Augeo Youth Task Force (a panel of young adults with CA; https://www.augeo.nl/jongerentaskforce), a group of young people with lived experiences of CA at multiple timepoints during all phases of the study. The Augeo Youth Task Force is part of the Augeo Foundation, a Dutch organisation with the mission to help ensure that children can grow up safely and healthily. The taskforce provides practical advice regarding the set-up, recruitment and analysis of the study, such as ways to improve participant retention in follow-up measurements, how to adjust communication strategies, and to improve recruitment materials. Collaborations with organisations like Augeo allow us to integrate the perspectives and priorities of young people with lived experience into the research design.

### Sample size estimation

To minimise attrition across the six assessment waves, retention strategies were developed in close collaboration with youth experts by experience from the Augeo Youth Task Force. These strategies include newsletters with study updates, a dedicated Instagram account, flexible scheduling of online questionnaires, friendly reminders and participant contact via WhatsApp in addition to email. Participation rates will be monitored throughout the study. Missing data patterns and potential predictors of missingness will be examined using baseline characteristics. Analyses will use statistical approaches that accommodate intermittent missing data, including full information maximum likelihood, allowing all available data to contribute to parameter estimation under standard missing-data assumptions.

We computed separate sample size estimations for each study objective. Our recruitment target is 288 participants, which includes a 20% oversampling to account for missing data and lower retention rates. In the power analyses, we therefore examined whether a sample size of 240 would be sufficient.

For objective 1, we simulated data based on the hypothesised dimensional model, assuming moderate effect sizes (β=0.3) from threat on emotion processing and deprivation on social feedback learning, with no other effects and a sample size of N=240 (this number is based on a 20% drop-out rate for the N=288 included participants). Model fit was then compared with that of a cumulative risk model, which assumes equal effects across domains. Using 1000 Monte Carlo simulations and the Bayesian Information Criterion to balance explanatory power and parsimony, we found that the hypothesised model was preferred in 99.8% of cases. The simulations also demonstrated >99.5% power to detect significant path coefficients for both key parameters.

Objective 2 examines the effects of CA, social support and their interaction on neural stress responses during the MIST. In a recent study from our group,^58^ we tested whether friendship quality was associated with reduced neural stress responses across five predefined ROIs in 102 young people with a history of CA. These analyses revealed only a small-to-medium buffering effect of friendship quality on left hippocampal reactivity, which did not survive correction for multiple comparisons. Based on these findings, we calculated the required sample size using G*Power (‘F test: multiple regression—fixed model, R² increase’). Assuming α=0.05, power=0.85, a small-to-medium main effect of CA and friendship support (f²=0.05; residual variance=0.95), and a 0.05 increase in explained variance through the interaction effect (M increase in R²=0.05 (variance explained by special effect=0.05)). The analysis indicated that a total sample of N=238 (f²=0.053) would be required.

Objective 3, we examined power for growth curve models using the SimSEM package.^85^ We evaluated both convergence rates and power to detect the effects of predictors (eg, emotion processing) on the intercept and slope under optimal (N=240, β>0.3) and high attrition (N=200, β>0.2) conditions. Under the optimal scenario, convergence was 100%, with power of 100% and 99.3% to detect slope variance and slope prediction, respectively. Under high attrition conditions, convergence remained 100%, with power of 100% and 67% for the same parameters.

For objective 4, we computed power for Random Intercept Cross-Lagged Panel Models using the powRICLPM package.^86^ These analyses tested whether within-person reductions in social support predicted within-person increases in mental health. Assuming an intraclass correlation coefficient of 0.4^87^ and a dynamic effect size of 0.25, we found 80% power to detect the effect of within-person changes in social support on mental health when N>200 and at least four repeated measures were available.

Taken together, these complementary power analyses show that our proposed sample size of 288 and 240 after allowing for 20% attrition provides sufficient power to detect the hypothesised effects across all study objectives.

## Ethics and dissemination

### Ethical approval

The study is conducted according to the principles of the Declaration of Helsinki^88^ and in accordance with the Dutch Medical Research Involving Human Subjects Act (WMO). This study was approved on 5 July 2022 by the Medical Ethics Committee Leiden The Hague Delft (NL80017.058.21). After this date, multiple minor and major amendments were approved. The protocol described here corresponds to the most recent amendment that was approved in May 2025.

### Ethical and safety considerations

All participants provide digital informed consent and get the opportunity to ask questions via email, telephone, mobile messages and in-person at the in-unit session. The information for participants also includes a global description of the stress inductions. Participants can discontinue the study at any time without explanation and unsubscribe via a form or by contacting the team directly. If they chose to do so, participants are reimbursed for the time they invested at that point. At the end of an in-unit session, trained team members thoroughly explain deception and manipulations used in the experimental tasks in a debriefing session. Team members check if participants understand the nature of deception and why the manipulations were implemented. Team members take the time to discuss participants’ experiences and feelings. In case a participant does experience emotional distress after the stress task, the debriefing can be brought forward. When participants experience emotional distress, team members address these feelings. Participants are offered a check-in call the next day when appropriate. All participants receive a list of mental health resources at the end of the in-unit session.

### Dissemination plan

The results of the THRIVE study will be disseminated through preprints, peer-reviewed scientific publications and scientific presentations, as well as through media and public outreach. The THRIVE study has been co-created with the Augeo Youth Task Force to ensure that the perspectives, priorities and lived experiences of young people with CA are meaningfully incorporated in study procedures, interpretation of findings and dissemination activities (see also the Patient and public involvement section).

We are committed to open access and transparency in our research. Publications will be submitted to open access journals where possible and archived, together with supporting documents, in a long-term open-access repository. Data preservation and publication will follow the FAIR principles.^89^ A data package of pseudonymised or anonymised research data will be deposited in DataverseNL in accordance with local archiving guidelines and made available on request. Where appropriate and feasible, study materials and analysis code may also be shared to support reproducibility.

To maximise societal impact, we will actively connect with relevant networks and stakeholders, including municipalities, knowledge partners and national organisations involved in mental health and youth well-being. We will also share findings within existing academic and practice-oriented consortia, such as the SSH Sectorplan Resilience in Youth consortium. In addition, we welcome opportunities for collaboration and knowledge exchange with national and international partners.

To make results accessible to participants, practitioners, policymakers and the wider public, we will communicate findings in multiple formats and levels of detail. This may include plain-language summaries, infographics, website and newsletter updates and broader outreach via social media and popular media. For any PhD theses and major outputs resulting from this project, we will also prepare accessible summaries of key findings to support dissemination beyond academia.

## Supplementary material

10.1136/bmjopen-2026-123790 online supplemental file 1
