# Supplementary material for: Longitudinal THRIVE study protocol: social and neurobiological mechanisms of risk and resilience in young people with a history of childhood adversity
Source: BMJ Open. 2026 Oct 29;16(10):e123790. doi: 10.1136/bmjopen-2026-123790 (PMC13723858; doi:10.1136/bmjopen-2026-123790)
Supplement: Supplementary file 1 [file bmjopen-2026-123790.reviewer_comments.pdf]

PEER REVIEW HISTORY

BMJ Open publishes all reviews undertaken for accepted manuscripts. Reviewers are asked to complete a checklist review form and are provided with free text boxes to elaborate on their assessment. These free text comments are reproduced below.

ARTICLE DETAILS

Title (Provisional)

The Longitudinal THRIVE study Protocol: Social and Neurobiological Mechanisms of Risk and Resilience in Young People with a History of Childhood Adversity

Authors

Buimer, Elizabeth EL; König, Maximilian; Wessels, Pauline; Dorsman, Hannah; Haverhals, Antoinette; Consortium, THRIVE; Bos, Peter A; Will, Geert-Jan; Harmelen , Anne-Laura van

---

VERSION 1 - REVIEW

**Reviewer**	**1**
**Name**	**Barnett, Whitney**
**Affiliation**	**University of Cape Town Department of Paediatrics and Child Health, Paediatrics & Child Health**
**Date**	**11-Aug-2026**
**COI**	**None**

This is an exceptionally interesting and well-motivated study, and I was particularly impressed by the Introduction, which provides a strong rationale for the study objectives and hypotheses. The protocol is generally very clear, and the figures and table summarizing the measures are especially helpful. My comments below are primarily intended to clarify the operationalization of childhood adversity and the planned analytic approach.

Abstract

• Given the breadth of the term “childhood adversity,” I suggest defining more clearly what is meant by it in this study. The examples currently provided (abuse, neglect, bullying) do not fully align with either traditional ACEs or the adversities assessed by the YCAS, and this also makes the stated 50% prevalence difficult to interpret. A brief definition early in the Abstract would help orient the reader.

Introduction/Methods

• Relatedly, I suggest greater consistency and precision in the use of childhood adversity versus adverse childhood experiences (ACEs) throughout. The “one out of two” estimate seems reasonable shorthand for the prevalence reported by Madigan et al., but poverty and bullying were not among the traditional ACEs included in that review, nor are they assessed by the YCAS. Please either revise

these examples or ensure that the cited prevalence estimate supports the broader definition being used.

• For the PHQ exclusion criterion (“total score higher than 14 or a score higher than 0 on the suicidal ideation item”), please briefly indicate what these thresholds represent for readers unfamiliar with the measure.

• Please clarify how the YCAS and CTQ are intended to complement one another. There appears to be overlap in some adversities assessed by the two measures, particularly abuse/violence. Are they intended to capture different developmental periods? If not, how will potentially overlapping endorsements be handled when information from both measures is combined?

• I would appreciate greater clarity regarding how the cumulative versus dimensional models in Objective 1 will be operationalized and compared. A cumulative adversity model would typically reflect the amount/breadth of adversity exposure irrespective of type, whereas PCA identifies components based on covariance among items. A PCA-derived component is therefore not necessarily equivalent to a cumulative adversity index, nor will empirically derived components necessarily correspond to the theoretically specified threat/deprivation dimensions. Please specify a priori how cumulative adversity, threat, and deprivation will each be operationalized and what model comparison will constitute support for one theoretical account over the other.

• Minor analytic point: PCA yields components rather than latent factors. If the goal is to identify latent dimensions underlying adversity items, please clarify the rationale for PCA rather than a latent factor approach (e.g., EFA).

• The sample-size simulations are helpful and the recruitment target appears well justified under the specified assumptions. Given the 6 assessments across approximately one year, however, please provide a little more detail regarding the plan for intermittent missing data and attrition. Will patterns/predictors of missingness be examined, and will recruitment be monitored or increased if attrition exceeds the anticipated 20%?

• The figures and table summarizing the measures are extremely helpful and make this relatively complex protocol much easier to follow.

VERSION 1 - AUTHOR RESPONSE

Reviewer: 1
Mrs. Whitney Barnett, University of Cape Town Department of Paediatrics and Child Health, Vanderbilt University

Comments to the Author:

This is an exceptionally interesting and well-motivated study, and I was particularly impressed by the Introduction, which provides a strong rationale for the study objectives and hypotheses. The protocol is generally very clear, and the figures and table summarizing the measures are especially helpful. My comments below are

primarily intended to clarify the operationalization of childhood adversity and the planned analytic approach.

We thank the reviewer for their positive evaluation of our manuscript. We appreciate the recognition of the study rationale, the clarity of the protocol, and the usefulness of the figures and table summarizing the measures. We also thank the reviewer for their constructive comments regarding the operationalization of childhood adversity and the planned analytical approach. We address each comment (in bold) below, and have copied the relevant excerpt from the revised manuscript (in italics) where revisions to this manuscript text are indicated in bold.

Abstract

1. **Given the breadth of the term "childhood adversity," I suggest defining more clearly what is meant by it in this study. The examples currently provided (abuse, neglect, bullying) do not fully align with either traditional ACEs or the adversities assessed by the YCAS, and this also makes the stated 50% prevalence difficult to interpret. A brief definition early in the Abstract would help orient the reader.**

We thank the reviewer for this observation. We agree that the examples provided and the cited prevalence were confusing. To avoid ambiguity, we now removed the prevalence from the abstract and provide a clear definition of childhood adversity instead.

Abstract

"Introduction

Childhood adversity (CA) is among the strongest predictors of mental health problems.

CA refers to experiences that are likely to require significant adaptation by an average

child and that represent a deviation from the expectable environment."

2. **Relatedly, I suggest greater consistency and precision in the use of childhood adversity versus adverse childhood experiences (ACEs) throughout. The "one out of two" estimate seems reasonable shorthand for the prevalence reported by Madigan et al., but poverty and bullying were not among the traditional ACEs included in that review, nor are they assessed by the YCAS. Please either revise these examples or ensure that the cited prevalence estimate supports the broader definition being used.**

Thank you for catching our error. We revised the sentence reporting ACE prevalence and now list only ACEs that were included in the cited prevalence study. Furthermore, we added a sentence acknowledging that prevalence rates vary across studies depending on the operationalization of childhood adversity and the assessment methods used. Finally, we clarified how childhood adversity was operationalized in the present study, namely as a broad construct encompassing abuse and neglect (measured using the CTQ) as well as other adverse experience that occurred in childhood or adolescence, such as parental separation and the loss of a loved one (measured using the YCAS).

Introduction

"One out of two children and adolescents growing up worldwide are exposed to at least one ***adverse childhood experience, such as abuse, witnessing domestic violence or substance abuse in the household [1]. Prevalence estimates of childhood adversity vary substantially across studies, depending on how adversity is conceptualized and measured, including the types of experiences assessed and the assessment methods used [2].*** *Childhood adversity can be defined as* ***"experiences that are likely to require significant adaptation by an average child and that represent a deviation from the expectable environment." [3, p. 363]****. As such, childhood adversity refers to a wide range of* ***adverse childhood*** *experiences including child maltreatment (emotional, sexual, and physical abuse, and emotional and physical neglect) as well as family adversity (e.g., marital distress/conflict, parental alcohol dependence, aggressive parenting behaviour, parental violence, parental mental health problems, or stressful family-level life events) [4].* ***The present study operationalizes childhood adversity as a broad construct encompassing abuse and neglect as well as other adverse experiences, such as parental separation and the loss of a loved one."***

3. **For the PHQ exclusion criterion ("total score higher than 14 or a score higher than 0 on the suicidal ideation item"), please briefly indicate what these thresholds represent for readers unfamiliar with the measure.**

We now clarified this exclusion criterion:

2. Methods and analysis
2.1 Sample recruitment and eligibility

"Given that the study protocol can be perceived as stressful, ***particularly for adolescents experiencing elevated levels of emotional distress,*** *we used the* ***9-item version of the*** *Patient Health Questionnaire [61] to assess severe depressive symptoms or suicidal ideation over the past two weeks****, rated on a Likert scale from 0 (not at all) to 3 (nearly every day)****. Young people were* ***excluded from participation if their*** *total score* ***was*** *higher than 14 or* ***when they scored*** *higher than 0 on the suicidal ideation item* ***("Thoughts that you would be better off dead or of hurting yourself in some way")****."*

4. **Please clarify how the YCAS and CTQ are intended to complement one another. There appears to be overlap in some adversities assessed by the two measures,**

particularly abuse/violence. Are they intended to capture different developmental periods? If not, how will potentially overlapping endorsements be handled when information from both measures is combined?

We utilized the CTQ and YCAS for different reasons. First, for our study we wanted to include individuals with a self-reported history of CA. Therefore, we choose to use the YCAS as a telephone screening instrument for study inclusion (i.e., participants must endorse at least one YCAS item). We choose the YCAS for this as it is a brief questionnaire, it captures a wide range of experiences, and it is formulated in such a way that it is not too stressful for individuals with lived experiences with CA to complete. We then added the CTQ to our study protocol as it is a well-validated instrument (Bernstein et al., 1994. *Am. J. Psychiatry*; Thombs et al., 2009. *Child Abuse Negl.*) for assessing specific forms of childhood maltreatment, (specifically abuse and neglect) that are not that well captured in the YCAS. Furthermore, the CTQ is a widely used instrument allowing for cross-cohort comparisons (Hagborg et al., 2022. *J. Child. Adolesc. Trauma*; MacDonald et al., 2016. *PloS one*).

For our analyses we use both measures to assess childhood adversity occurring before the age of 18 (and they are thus not intended to capture different developmental periods). There is some potential overlap between the two measures on two domains (i.e. exposure to violence and traumatic sexual experiences). However, the items in the CTQ are strongly contextualized within the home environment, and these experiences can also happen outside the home environment. As a result, the YCAS may identify experiences that would not be captured by the CTQ. Thus, the YCAS captures adverse experiences that may occur both within and outside the family context, whereas the CTQ primarily focuses on abuse and neglect within the familial environment.

When data from both measures are combined in the analyses, we will take into account potential covariance between these items. For instance, our principle component data reduction technique is chosen as this will ensure that highly related or overlapping indicators load on to the same component rather than being treated as distinct adversity domains. This information is now added in the manuscript.

<u>Methods</u>

<u>2.4 Data analysis</u>

"2.4.1 ***Data reduction methods to*** *estimate* ***main*** *constructs*

To estimate our ***main*** *constructs (****i.e.,*** *childhood adversity, social support, social stress, and mental health), we will use Principal Component Analyses (PCA)* ***as a data reduction technique. Separate PCAs will be conducted for each core construct, using item-level data from the questionnaires that assess that specific construct. This approach allows us to derive component scores that summarize patterns of covariation across items measuring the same underlying construct. For childhood adversity, the Childhood***

Trauma Questionnaire was included to provide a detailed assessment of abuse and neglect within the family context, while the Youth and Childhood Adversity Scale was used to capture a broader range of adverse childhood experiences occurring both inside and outside the family context. Items from both questionnaires will be entered into the PCA for childhood adversity, thereby accounting for covariance between items across the two measures."

5. **I would appreciate greater clarity regarding how the cumulative versus dimensional models in Objective 1 will be operationalized and compared. A cumulative adversity model would typically reflect the amount/breadth of adversity exposure irrespective of type, whereas PCA identifies components based on covariance among items. A PCA-derived component is therefore not necessarily equivalent to a cumulative adversity index, nor will empirically derived components necessarily correspond to the theoretically specified threat/deprivation dimensions. Please specify a priori how cumulative adversity, threat, and deprivation will each be operationalized and what model comparison will constitute support for one theoretical account over the other.**

We appreciate this important point. To clarify, Objective 1 will not estimate a specific cumulative adversity score. We will estimate threat and deprivation components based on the Childhood Trauma Questionnaire. Consistent with previous work (e.g., Lambert et al., 2016. *Dev. Psychopathol.*; Liuzzi et al., 2024. *Biol. Psychiatry: Cogn. Neurosci. Neuroimaging*), deprivation will be indexed by the emotional and physical neglect subscales, whereas threat will be indexed by the emotional, physical, and sexual abuse subscales.

We will then utilize path modelling using these two dimensions as predictors to evaluate whether our model suggests that these two dimensions have similar (which would support accumulation models) or distinct (which would support dimensional models) relationships with neurocognitive functioning (i.e. threat related information processing and feedback learning).

Specifically, dimensional models suggest that experiences of threat are hypothesized to predict altered threat-related information processing, whereas deprivation experiences are hypothesized to predict altered feedback learning. In contrast, cumulative risk models do not assume that the type of adversity has unique contributions and would thus assume equivalent contributions between deprivation and threat with neurocognitive outcomes.

Operationally, we will examine in our path model whether the paths between threat and deprivation to threat related information processing and feedback learning are similar or distinct. We will do so by constraining the paths from threat and deprivation to threat related information processing and feedback learning to be equal in magnitude and to then compare this to a model where these paths differ.

Evidence for the cumulative risk account would therefore be provided if a more parsimonious model, in which threat and deprivation have equivalent effects across outcomes, fits the data

as well as, or better than, a model allowing distinct effects. Conversely, evidence for the dimensional account would be provided if allowing threat and deprivation to have unique associations with different outcomes significantly improves model fit.

Thus, the comparison is not between dimensional scores and a cumulative adversity index. Our findings will be based on the comparison of differential pathmodels (using the exact same predictors and outcomes, whilst varying their inter-relationships). The results of these model comparisons will provide evidence for one of the two competing theoretical models. If the model suggests unique effects of threat and deprivation on neurocognitive functioning outcomes this would be in line with dimensional models, however if our model suggests equivalent effects this would be more consistent with a cumulative risk framework.

We have now clarified the operationalisation, model comparison approach, and rationale in the manuscript. References to cumulative risk index in this context are removed. We also identified and corrected an error in Figure 1 related to Objective 1.

<u>Introduction</u>

"The THRIVE study has four objectives (Figure 1). As a first objective, we will compare two competing hypotheses ***regarding the effects of childhood adversity on emotion processing and social feedback learning. Specifically, we will investigate whether associations with these outcomes are best explained by distinct effects of threat and deprivation experiences (dimensional approach) or by equivalent, non-specific effects across adversity dimensions (cumulative risk approach).*** *Based on the dimensional model [16], we expect that threat experiences predict biased information processing towards threat cues in an emotion processing task but not blunted social feedback learning. In contrast, deprivation experiences would result in blunted social feedback learning but not biased information processing. Support for the competing cumulative* ***risk account*** *would be found when threat and deprivation experiences* ***show similar effects on both emotion processing and feedback learning outcomes, suggesting that the overall burden of adversity is more important than the specific nature of adversity exposure."***

Figure 1 *The main objectives of the THRIVE study*.

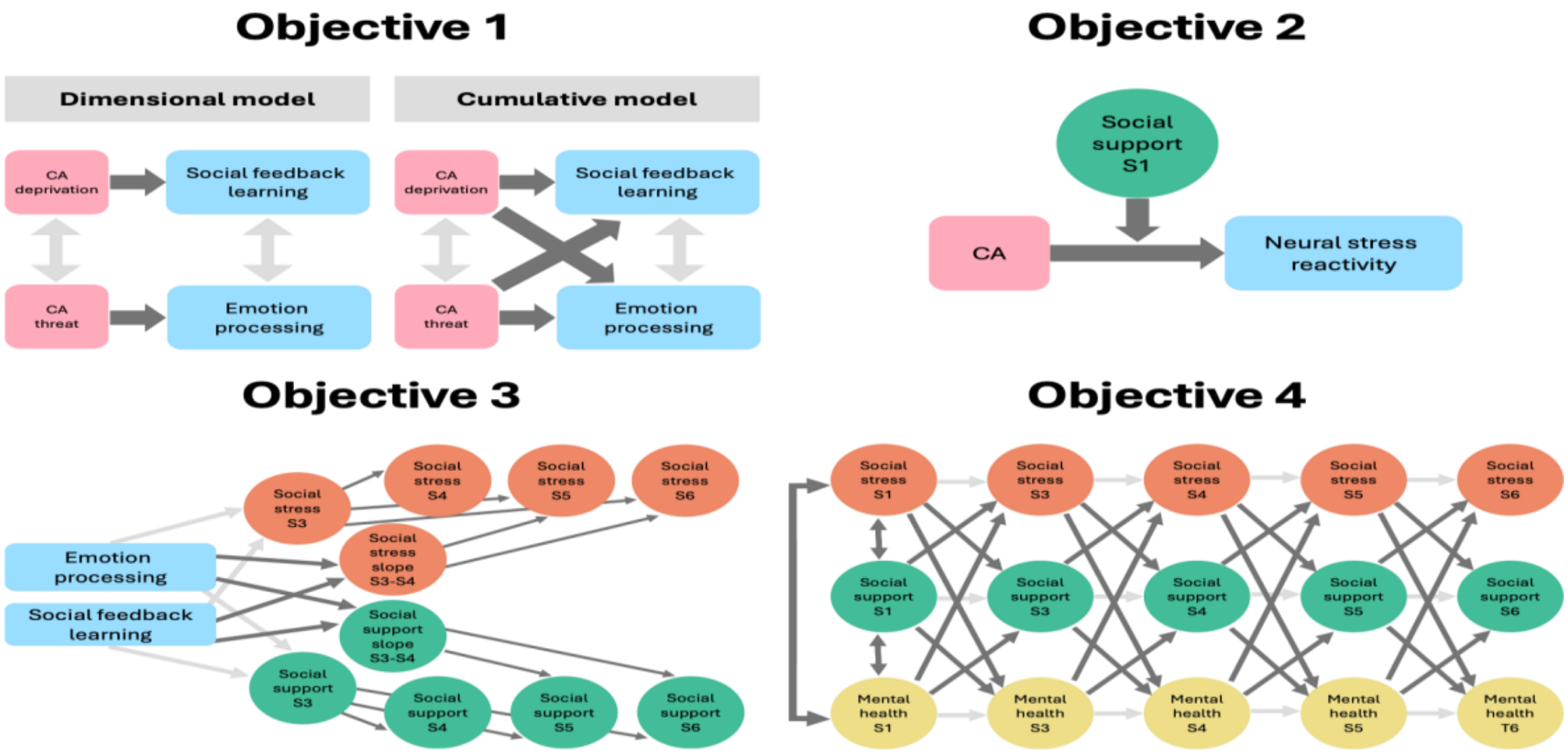

Note: The THRIVE study tests four objectives: (1) **Utilizing path modelling and model comparisons to evaluate evidence for dimensional and cumulative models of childhood adversity (CA). Model comparisons will be used to examine whether threat and deprivation have distinct effects on emotion processing (threat bias) and social feedback learning (blunting). Distinct effects would support dimensional models of CA, whereas equivalent effects would support cumulative risk models.** (2) Social stress buffering hypothesis: Testing whether social support moderates the association between CA and neural stress reactivity. (3) Social transactional model: Using longitudinal assessments (S1 to S6) to evaluate whether CA-related alterations in emotion processing and feedback learning predict stress generation and social thinning (more stressful experiences, lower social support, and steeper worsening over time). (4) Within-person dynamics: Modelling intra-individual coupling between friendship quality, social stress, and mental health, hypothesizing that within-person reductions in friendship quality and increases in social stress precede declines in mental health. S1 to S6 refer to the different sessions at which the repeated measures are collected.

Methods

2.4 Data analysis

2.4.1 **Data reduction methods to** estimate **main** constructs

"To compare dimensional and cumulative risk accounts (Objective 1), we will derive threat and deprivation scores based on theoretically defined adversity categories. Consistent with previous work [102-103], deprivation will be indexed by emotional and physical neglect experiences, whereas threat will be indexed by emotional, physical, and sexual abuse experiences. These threat and deprivation scores will then be included and using path modelling we will then evaluate their relations with neurocognitive functioning using model comparisons. In the dimensional path-model, threat and deprivation will be allowed to show distinct associations with emotion processing and social feedback learning outcomes. In the cumulative risk path-model, paths from threat and deprivation to the outcomes will be constrained to be equal, reflecting the hypothesis that adversity

dimensions exert comparable, non-specific effects. Model fit indices and parsimony criteria will be used to determine which theoretical account better explains the data."

6. **Minor analytic point: PCA yields components rather than latent factors. If the goal is to identify latent dimensions underlying adversity items, please clarify the rationale for PCA rather than a latent factor approach (e.g., EFA).**

We fully agree with the reviewer that latent factors should not be used interchangeably with components. We would like to clarify that our aim was not to identify latent factors that underlie childhood adversity items as we do not conceptualize childhood adversity as a latent construct that gives rise to the observed adverse experiences. Instead, we will use Principal Component Analysis (PCA) as data reduction technique to summarize patterns of covarying adversities to reduce the number of variables included in our subsequent analyses.

To avoid confusion regarding the interpretation of the PCA results, we have revised the manuscript throughout and removed references to "latent factors". We now consistently refer to the PCA-derived components as components. We have also clarified the rationale for using PCA in the Methods section, emphasizing its role as a data reduction method rather than a latent variable approach such as exploratory or confirmatory factor analysis.

<u>Methods</u>

<u>2.4 Data analysis</u>

"2.4.1 ***Data reduction methods to*** *estimate* ***main*** *constructs*

To estimate our ***main*** *constructs (****i.e.,*** *childhood adversity, social support, social stress, and mental health), we will use Principal Component Analyses (PCA)* ***as a data reduction technique. Separate PCAs will be conducted for each core construct, using item-level data from the questionnaires that assess that specific construct. This approach allows us to derive component scores that summarize patterns of covariation across items measuring the same underlying construct. For childhood adversity, the Childhood Trauma Questionnaire was included to provide a detailed assessment of abuse and neglect within the family context, while the Youth and Childhood Adversity Scale was used to capture a broader range of adverse childhood experiences occurring both inside and outside the family context. Items from both questionnaires will be entered into the PCA for childhood adversity, thereby accounting for covariance between items across the two measures."***

"2.4.2 Residual approach to quantify resilient functioning

Resilient functioning will be quantified as the degree to which an individual functions better or worse than expected given their self-reported history of childhood adversity [53]. For that, we will use PCAs that reflect current psychosocial functioning across various domains (i.e., assessed with the Drugs, Alcohol, and Self-Injury Inventory, Mood and Feelings Questionnaire, State-Trait Anxiety Inventory, Patient Health Questionnaire) as well as a single ***component*** *that reflects general childhood adversity experiences (assessed with the Childhood Trauma Questionnaire and Youth and Childhood Adversity Scale). Individual degree of resilient functioning will then be estimated as the residual variation from the best fitting relationship (comparing linear, quadratic, and cubic models) between our* ***component*** *for psychosocial functioning and our* ***component*** *for childhood adversity."*

7. **The sample-size simulations are helpful and the recruitment target appears well justified under the specified assumptions. Given the 6 assessments across approximately one year, however, please provide a little more detail regarding the plan for intermittent missing data and attrition. Will patterns/predictors of missingness be examined, and will recruitment be monitored or increased if attrition exceeds the anticipated 20%?**

We thank the reviewer for pointing us to this missing information. We will closely monitor participation rates throughout the study and continue to use participant engagement strategies to encourage participant retention across all assessment waves. Furthermore, we implemented several strategies to minimise intermittent missing data and attrition throughout the study. These retention procedures were developed in close collaboration with youth experts by experience from the Augeo Youth Task Force to ensure that study procedures and communication remain engaging and appropriate for young participants. Strategies include newsletters with study updates, a dedicated Instagram account, flexible scheduling of online questionnaires, friendly reminders, and participant contact via WhatsApp in addition to email.

We expect some degree of intermittent missingness and attrition, which is inevitable in longitudinal research with multiple assessment waves. Based on our ongoing data collection, attrition rates have thus far been relatively low. For example, of the 283 enrolled participants, 243 completed both the first and second assessment, and 206 participants have completed the third assessment to date. The small number of dropouts was primarily attributable to practical circumstances rather than concerns related to study participation.

To assess the possibility of systematic attrition, we will examine patterns and predictors of missingness by comparing participants with complete versus incomplete follow-up data on relevant baseline characteristics. Analyses will use statistical approaches that accommodate intermittent missing data, including Full Information Maximum Likelihood

(FIML), allowing all available data to contribute to parameter estimation under standard missing-data assumptions.

We do not have ethics to increase recruitment if attrition exceeds the anticipated 20%. The reason for this is that our target sample size was determined a priori through power analyses using simulations that incorporated an expected attrition of 20%. Therefore, our strategy focuses on maximising participant retention and using appropriate statistical methods to handle intermittent missing data.

<u>Methods</u>

“2.6 Sample size estimation

To minimise attrition across the six assessment waves, retention strategies were developed in close collaboration with youth experts by experience from the Augeo Youth Task Force. These strategies include newsletters with study updates, a dedicated Instagram account, flexible scheduling of online questionnaires, friendly reminders, and participant contact via WhatsApp in addition to email.
Participation rates will be monitored throughout the study. Missing data patterns and potential predictors of missingness will be examined using baseline characteristics. Analyses will use statistical approaches that accommodate intermittent missing data, including Full Information Maximum Likelihood, allowing all available data to contribute to parameter estimation under standard missing-data assumptions.”

8. **The figures and table summarizing the measures are extremely helpful and make this relatively complex protocol much easier to follow.**

Thank you for this encouraging comment. We are glad that the figures and table help present the protocol in a clear and accessible manner, given the complexity of the study design.

VERSION 2 - REVIEW

Reviewer 1

Name Barnett, Whitney

Affiliation University of Cape Town Department of Paediatrics and Child Health, Paediatrics & Child Health

Date 06-Oct-2026

COI

The authors have addressed my previous comments thoroughly, and I have no further substantive concerns. One minor inconsistency to correct: the Methods now state that recruitment closed with a final sample of 283 participants, but the Abstract still describes the study as aiming to recruit 288, and the first Strengths and Limitations bullet states that 288 young adults "will be included." I think it is helpful to reconcile this to avoid confusion. I am accepting this manuscript so this is a suggestion for clarity.

VERSION 2 - AUTHOR RESPONSE

Reviewer: 1

Mrs. Whitney Barnett, University of Cape Town Department of Paediatrics and Child Health, Vanderbilt University

Comments to the Author:

The authors have addressed my previous comments thoroughly, and I have no further substantive concerns. One minor inconsistency to correct: the Methods now state that recruitment closed with a final sample of 283 participants, but the Abstract still describes the study as aiming to recruit 288, and the first Strengths and Limitations bullet states that 288 young adults "will be included." I think it is helpful to reconcile this to avoid confusion. I am accepting this manuscript so this is a suggestion for clarity.

Author's reply:

We thank the reviewer for the positive evaluation of the revised manuscript. The inconsistencies identified by the reviewer have now been corrected. The Abstract and the Strengths and Limitations sections have been updated to reflect the correct sample size:

Abstract

Methods and analysis

"The ongoing THRIVE study includes 283 participants aged 18-24 years who retrospectively self-report CA."

Strengths and limitations of this study

● "A large population of 283 young adults with self-reported childhood adversity is included."
