# Supplementary material for: Longitudinal THRIVE study protocol: social and neurobiological mechanisms of risk and resilience in young people with a history of childhood adversity
Source: BMJ Open. 2026 Oct 29;16(10):e123790. doi: 10.1136/bmjopen-2026-123790 (PMC13723858; doi:10.1136/bmjopen-2026-123790)
Supplement: Supplementary file 2 [file bmjopen-16-10-s001.docx]

Social and Neurobiological Mechanisms of Risk and Resilience in Young People: The Longitudinal THRIVE study

*Running title:* The THRIVE study protocol

Elizabeth E.L. Buimer^1,2*^, Maximilian König^1,2*^, Pauline Wessels^1,2^, Hannah Dorsman^1,2^, Antoinette Haverhals^1,2^, THRIVE consortium^3^, Peter A. Bos^1^, Geert-Jan Will^2,4^ & Anne-Laura van Harmelen^1,2+^.

^1^ Institute of Education and Child Studies, Leiden University, Leiden, The Netherlands.

^2^ Leiden Institute for Brain and Cognition, Leiden University Medical Center, Leiden, The Netherlands

^3^ Members of the THRIVE consortium and their affiliations are listed in the collaborators section.

^4^ Department of Clinical Psychology, Utrecht University, Utrecht, the Netherlands

^*^ Both authors contributed equally.

^+^ corresponding authors: [a.van.harmelen@fsw.leidenuniv.nl](mailto:a.van.harmelen@fsw.leidenuniv.nl)

# Supplement

## S.1 Additional information on the questionnaires used in the THRIVE study.

### S1.1 CHILDHOOD ADVERSITY

#### Youth and Childhood Adversity Scale (YCAS)

The Youth and Childhood Adversity Scale (Schlechter et al., 2021) is a standardized, retrospective 13-item self-report inventory that measures the severity of different types of adverse experiences (e.g., serious illness, serious parental illness, or “other” adversity). If participants endorse an “other” adverse event they will be given an open-ended option to specify this event in further detail. Participants indicate if they experienced each event before the age of 18 (dichotomous yes/no) and how traumatic the event was in their experience (7-point scale from 1 = “not at all traumatic” to 7 = “extremely traumatic”). This questionnaire takes approximately 2-minutes to complete.

#### Childhood Trauma Questionnaire (CTQ)

The Childhood Trauma Questionnaire (Bernstein et al., 1994) is a standardized, retrospective 28-item self-report inventory that measures the severity of different types of childhood trauma, producing five subscales: emotional abuse, physical abuse, sexual abuse, emotional neglect, and physical neglect. The measure also includes a 3-item minimization/denial scale indicating the potential underreporting of maltreatment. We used the Dutch version which has 5 instead of 4 items for the sexual abuse subscale, resulting in a total of 27 items (Thombs et al., 2009). Participants respond to each item in the context of “when you were growing up” and answer according to a 5-point Likert scale ranging from 1 = “never” to 5 = “very often”. This questionnaire takes approximately 3-minutes to complete.

### S1.2 MENTAL HEALTH

#### Patient Health Questionnaire (PHQ)

The Patient Health Questionnaire (Kroenke et al., 2001) is a 9-item self-report inventory assessing levels of depressive symptoms. Participants respond to each item on a 4-point Likert scale ranging from 1 = “not at all” to 4 = “nearly every day”. This questionnaire takes approximately 2-minutes to complete.

#### State-Trait Anxiety Inventory (STAI)

The State-Trait Anxiety Inventory (Spielberger et al., 1983) is a 40-item self-report inventory assessing trait and state anxiety. Participants respond to state anxiety items in the context of “how you feel right now, that is at this moment” on a 4-point Likert scale ranging from 1 = “not at all” to 4 = “very much so”. Responses to trait anxiety items will happen in the context of “how you generally feel” again on a 4-point Likert scale ranging from 1 = “almost never” to 4 = “almost always”. This questionnaire takes approximately 3-minutes to complete.

#### Mood and Feelings Questionnaire (MFQ)

The Mood and Feelings Questionnaire (Angold et al., 1995) is a 33-item self-report inventory assessing depressive symptoms. Participants respond to items in the context of “in the past two weeks” on a 3-point Likert scale ranging from 1 = “not true” to 3 = “true”. This questionnaire takes 2-minutes to complete.

#### Drugs, Alcohol and Self-Injury Inventory (DASI)

The Drugs, Alcohol, and Self-Injury Inventory (Cassels et al., 2018) is a 14-item self-report inventory assessing cigarette, alcohol, drug use, and non-suicidal self-injury. Participants respond to items on either a 4-point Likert scale ranging from 1 = “never” to 4 = “every day or nearly every day”, respond to items with “yes” or “no”, or provide responses in open text fields. This questionnaire takes approximately 3-minutes to complete.

#### Psychiatric History Self-Report Questionnaire (PHSQ)

The Psychiatric History Self-Report Questionnaire consists of four multiple choice questions assessing an individual’s history of current and lifetime psychiatric diagnoses, as well as current treatment(s) for mental health problems. This questionnaire takes approximately 1 minute to complete.

### S1.3 SOCIAL SUPPORT

#### McGill Friendship Questionnaire – Friendship Functions (MFQ-FF)

The McGill Friendship Questionnaire – Friendship Functions (Mendelson & Aboud, 1999) is a 30-item self-report inventory assessing perceived friendship support across six subscales: stimulating companionship, help, intimacy, reliable alliance, selfvalidation, and emotional security. Participants are asked to rate how their friend behaves towards them on a 5-point Likert scale ranging from 1 = “never” to 5 = “always”. This questionnaire takes approximately 4-minutes to complete.

#### Multidimensional Scale of Perceived Social Support (MSPSS)

The Multidimensional Scale of Perceived Social Support (Zimet et al., 1990) is a 12-item self-report inventory assessing social support across three domains: friends, family, and significant other. Participants respond to each item in the context of how they feel about specific statements on a 7-point Likert scale ranging from 1 = “very strongly disagree” to 7 = “very strongly agree”. This questionnaire takes approximately 1-minute to complete.

#### Cambridge Friendship Questionnaire (CFQ)

The Cambridge Friendship Questionnaire (van Harmelen et al., 2016, 2017) is an 8-item self-report inventory assessing the number, availability, and quality of friendships. Participants respond to each item in the context of “how they feel about their friendships” across changing scales. This questionnaire takes approximately 3-minutes to complete.

#### Strengths and Difficulties Questionnaire (SDQ)

The Strengths and Difficulties Questionnaire (Goodman 1997; Goodman, 2001) is a screening questionnaire to assess problem behavior in children. There is an adapted version available for adult self-report that is also translated to Dutch. To assess the construct social support, we will use the prosocial behavior (SDQ-ps) subscale which consists of 5 items and a 3-point Likert scale (“Not true” / “Partly true” / “Very true”). This questionnaire takes approximately 1 minute to complete.

### S1.4 SOCIAL STRESS

#### Multidimensional Peer Victimization Scale (MPVS)

The Multidimensional Peer Victimization Scale (Joseph & Stockton, 2018) is a 24-item self-report inventory assessing multiple facets of peer victimization with six subscales: physical victimization, verbal victimization, social manipulation, attacks on property, electronic victimization and social rebuff. Participants respond to each item in the context of “during the past year” and answer according to a 3-point Likert scale ranging from 1 = “not at all” to 3 = “more than once”. This questionnaire takes approximately 3 minutes to complete.

#### Life Events Questionnaire (LEQ)

This questionnaire consists of a 24-item list of life events. The questionnaire is based on lists used in other studies. We included items from studies that distinguish between dependent and independent items (Masten et al., 1994; Skaug et al., 2022) or focused on longitudinal mapping of stressors for predictions (Bergman et al., 2007; Wang et al., 2015). We also incorporated items used in large population-based cohorts, such as the ABCD study (Tiet et al., 1998) and the YOUth cohort study (Buimer et al., 2022). Participants are asked to indicate if one of the events occurred in the past three months and what the impact of this event was on a 7-point Likert scale ranging from 1 = “not stressful at all” to 7 = “extremely stressful”. Items from different studies were combined to create a list that is age-appropriate (matches adolescents), focuses on recent events (not childhood events or chronic stressors) and contains items that are dependent on the individual (stress generation). For the latter we relied on a list of dependent negative life events by Masten et al., 1994. Additionally, some items were added based on feedback from Dutch peers (for example, rejected for a room in a student house by prospective roommates). This questionnaire takes approximately 5 minutes to complete.

#### Strengths and Difficulties Questionnaire (SDQ)

The Strengths and Difficulties Questionnaire (Goodman 1997; Goodman, 2001) is a screening questionnaire to assess problem behavior in children. There is an adapted version available for adult self-report that is also translated to Dutch. To assess the construct social stress, we will use the peer problems subscale which consists of 5 items and a 3-point Likert scale (“Not true” / “Partly true” / “Very true”). This questionnaire takes approximately 1 minute to complete.

#### Perceived Stress Scale (PSS)

The Perceived Stress Scale (Cohen et al., 1983) is a 10-item self-report inventory assessing individual stress levels. Participants respond to each item in the context of their “feelings and thoughts during the last month” on a 5-point Likert scale ranging from 1 = “never” to 5 = “very often”. This questionnaire takes approximately 2 minutes to complete.

### S1.5 PERSONAL TRAITS AND SELF-ESTEEM

#### The Big Five Inventory-2 (BFI-2)

The Big Five Inventory-2 (Soto & John, 2017) is a 60-item self-report inventory assessing the Big Five traits: extraversion, agreeableness, conscientiousness, neuroticism, intellect/imagination. Due to time constraints, we will only assess 24-items related to extraversion and negative emotionality (traditionally known as neuroticism). Participants respond to each item on a 5-point Likert scale ranging from 1 = “disagree strongly” to 5 = “agree strongly”. This questionnaire takes approximately 5 minutes to complete.

#### Rosenberg Self-Esteem Scale (RSES)

The Rosenberg Self-Esteem Scale (Rosenberg, 1965) is a 10-item self-report inventory to assess positive and negative feelings of self-worth. Participants respond to each item on a 4-point Likert scale ranging from 1 = “strongly agree” to 4 = “strongly disagree”. This questionnaire takes approximately 2 minutes to complete.

#### Hypomanic Personality Scale (HPS)

The International Personality Item Pool version of the Hypomanic Personality Scale (Eckblad & Chapman, 1986) is a 12-item self-report inventory assessing an overactive, gregarious personality style that has been shown to correlate with emotional instability, as well as risk for developing bipolar disorder. Participants respond to each item on a 5-point Likert scale ranging from 1 = “very inaccurate” to 5 = “very accurate”. This questionnaire takes approximately 2 minutes to complete.

### S1.6 OTHER

#### Social Media Use Questionnaire (SMUQ)

The Social Media Use Questionnaire (Ellison et al., 2007) is a 8-item self-report inventory assessing social media use. Participants respond to two items on a 6-point Likert scale ranging from 1 = “less than 10 minutes” to 6 = “more than 3 hours” and to the remaining six items on a 5-point Likert scale ranging from 1 = “strongly disagree” to 5 = “strongly agree”. This questionnaire takes approximately 2 minutes to complete.

#### Coronavirus Impact Scale (CIS)

The Coronavirus Impact Scale (Stoddard et al., 2021) is a 12-item self-report inventory assessing multiple aspects of COVID-19 related impacts on individuals. Participants respond to each item in the context of “how much the COVID-19 pandemic has changed their life” on a 4-point Likert scale ranging from 1 = “no change” to 4 = “severe”. The final item is a free-response item. This questionnaire takes approximately 2 minutes to complete.

#### Edinburgh Handedness Inventory - Short Form (EHI-S)

The Edinburgh Handedness Inventory - Short Form (Veale, 2014) is a 4-items self-report inventory assessing handedness. Participants respond to each item on a 5- point Likert scale ranging from 1 = “always right” to 5 = “always left”. This questionnaire takes less than 1 minute to complete.

### References

Angold, A., Costello, E. J., Messer, S. C., & Pickles, A. (1995). Development of a short questionnaire for use in epidemiological studies of depression in children and adolescents. International journal of methods in psychiatric research.

Bergman, K., Sarkar, P., O'CONNOR, T. G., Modi, N., & Glover, V. (2007). Maternal stress during pregnancy predicts cognitive ability and fearfulness in infancy. Journal of the American Academy of Child & Adolescent Psychiatry, 46(11), 1454-1463. doi: <https://doi.org/10.1097/chi.0b013e31814a62f6>

Bernstein, D. P., Fink, L., Handelsman, L., Foote, J., Lovejoy, M., Wenzel, K., … Ruggiero, J. (1994). Initial reliability and validity of a new retrospective measure of child abuse and neglect. The American Journal of Psychiatry, 151(8), 1132–1136. doi: <http://doi.org/10.1176/ajp.151.8.1132>

Buimer, E. E.L., Brouwer, R. M., Mandl, R. C., Pas, P., Schnack, H. G., & Hulshoff Pol, H. E. (2022). Adverse childhood experiences and fronto-subcortical structures in the developing brain. Frontiers in psychiatry, 13, 955871. doi: <https://doi.org/10.3389/fpsyt.2022.955871>

Cassels, M., van Harmelen, A.-L., Neufeld, S., Goodyer, I., Jones, P. B., & Wilkinson, P. (2018). Poor family functioning mediates the link between childhood adversity and adolescent nonsuicidal self-injury. Journal of Child Psychology and Psychiatry, and Allied Disciplines, 59(8), 881–887. doi: <http://doi.org/10.1111/jcpp.12866>

Cohen, S., Kamarck, T., & Mermelstein, R. (1983). A global measure of perceived stress. Journal of health and social behavior, 385-396. doi: <https://doi.org/10.2307/2136404>

Ellison, N. B., Steinfield, C., & Lampe, C. (2007). The Benefits of Facebook “Friends:” Social Capital and College Students’ Use of Online Social Network Sites. Journal of Computer-Mediated Communication: JCMC, 12(4), 1143–1168. doi: <http://doi.org/10.1111/j.1083-6101.2007.00367.x>

Eckblad, M., & Chapman, L. J. (1986). Development and validation of a scale for hypomanic personality. Journal of Abnormal Psychology, 95(3), 214–222. doi: <http://doi.org/10.1037//0021-843x.95.3.214>

Goodman, R. (1997). The Strengths and Difficulties Questionnaire: a research note. Journal of child psychology and psychiatry, 38(5), 581-586. doi: <https://doi.org/10.1111/j.1469-7610.1997.tb01545.x>

Goodman, R. (2001). Psychometric properties of the strengths and difficulties questionnaire. Journal of the American Academy of Child & Adolescent Psychiatry, 40(11), 1337-1345. doi: <https://doi.org/10.1097/00004583-200111000-00015>

Joseph, S., & Stockton, H. (2018). The multidimensional peer victimization scale: A systematic review. Aggression and Violent Behavior, 42, 96–114. doi: <http://doi.org/10.1016/j.avb.2018.07.009>

Kroenke, K., Spitzer, R. L., & Williams, J. B. W. (2001). The PHQ-9. Journal of General Internal Medicine, 16(9), 606–613. doi: <http://doi.org/10.1046/j.1525-1497.2001.016009606.x>

Masten, A. S., Neemann, J., & Andenas, S. (1994). Life events and adjustment in adolescents: The significance of event independence, desirability, and chronicity. Journal of Research on Adolescence, 4(1), 71-97.

Mendelson, M. J., & Aboud, F. E. (1999). Measuring friendship quality in late adolescents and young adults: McGill Friendship Questionnaires. Canadian Journal of Behavioural Science. Revue Canadienne Des Sciences Du Comportement, 31(2), 130–132. doi: <http://doi.org/10.1037/h0087080>

Rosenberg, M. (1965). Rosenberg self-esteem scale (RSE). Acceptance and Commitment Therapy. Measures Package, 61(52), 18. doi: <https://psycnet.apa.org/doi/10.1037/t01038-000>

Schlechter, P., Fritz, J., Cassels, M., Neufeld, S. A. S., & Wilkinson, P. O. (2021). The Youth and Childhood Adversity Scale: a step towards developing a new measure of adversity and its severity. European Journal of Psychotraumatology, 12(1), 1981573. doi: <http://doi.org/10.1080/20008198.2021.1981573>

Skaug, E., Czajkowski, N. O., Waaktaar, T., & Torgersen, S. (2022). The relationship between life events and sense of coherence in adolescence. A longitudinal twin study. Journal of Research in Personality, 99, 104259. doi: <https://doi.org/10.1016/j.jrp.2022.104259>

Soto, C. J., & John, O. P. (2017). The next Big Five Inventory (BFI-2): Developing and assessing a hierarchical model with 15 facets to enhance bandwidth, fidelity, and predictive power. Journal of Personality and Social Psychology, 113(1), 117–143. doi: <http://doi.org/10.1037/pspp0000096>

Spielberger, C. D., Gorsuch, R. L., Lushene, R., Vagg, P. R., & Jacobs, G. A. (1983). Consulting Psychologists Press; Palo Alto, CA: 1983. Manual for the State-Trait Anxiety Inventory.

Stoddard, J., Reynolds, E., Paris, R., Haller, S. P., Johnson, S. B., Zik, J., ... & Kaufman, J. (2023). The coronavirus impact scale: construction, validation, and comparisons in diverse clinical samples. JAACAP open, 1(1), 48-59.

Thombs, B. D., Bernstein, D. P., Lobbestael, J., & Arntz, A. (2009). A validation study of the Dutch Childhood Trauma Questionnaire-Short Form: factor structure, reliability, and known-groups validity. Child abuse & neglect, 33(8), 518-523. doi: <https://doi.org/10.1016/j.chiabu.2009.03.001>

Tiet, Q. Q., Bird, H. R., Davies, M., Hoven, C., Cohen, P., Jensen, P. S., & Goodman, S. (1998). Adverse life events and resilience. Journal of the American Academy of Child & Adolescent Psychiatry, 37(11), 1191-1200. doi: <https://doi.org/10.1097/00004583-199811000-00020>

van Harmelen, Anne-Laura, Gibson, J. L., St Clair, M. C., Owens, M., Brodbeck, J., Dunn, V., … Goodyer, I. M. (2016). Friendships and Family Support Reduce Subsequent Depressive Symptoms in At-Risk Adolescents. PloS One, 11(5), e0153715. doi: <http://doi.org/10.1371/journal.pone.0153715>

van Harmelen, Anne-Laura, Kievit, R. A., Ioannidis, K., Neufeld, S., Jones, P. B., Bullmore, E., … Goodyer, I. (2017). Adolescent friendships predict later resilient functioning across psychosocial domains in a healthy community cohort. Psychological Medicine, 47(13), 2312–2322. doi: <http://doi.org/10.1017/S0033291717000836>

Veale, J. F. (2014). Edinburgh Handedness Inventory - Short Form: a revised version based on confirmatory factor analysis. Laterality, 19(2), 164–177. doi: <http://doi.org/10.1080/1357650X.2013.783045>

Wang, Y., Sareen, J., Afifi, T. O., Bolton, S. L., Johnson, E. A., & Bolton, J. M. (2015). A population-based longitudinal study of recent stressful life events as risk factors for suicidal behavior in major depressive disorder. Archives of suicide research, 19(2), 202-217. doi: <https://doi.org/10.1080/13811118.2014.957448>

Zimet, G. D., Powell, S. S., Farley, G. K., Werkman, S., & Berkoff, K. A. (1990). Psychometric characteristics of the Multidimensional Scale of Perceived Social Support. Journal of Personality Assessment, 55(3–4), 610–617. doi: <http://doi.org/10.1080/00223891.1990.9674095>
